# The homeodomain of Oct4 is a dimeric binder of methylated CpG elements

**DOI:** 10.1093/nar/gkac1262

**Published:** 2023-01-12

**Authors:** Daisylyn Senna Tan, Shun Lai Cheung, Ya Gao, Maike Weinbuch, Haoqing Hu, Liyang Shi, Shih-Chieh Ti, Andrew P Hutchins, Vlad Cojocaru, Ralf Jauch

**Affiliations:** School of Biomedical Sciences, Li Ka Shing Faculty of Medicine, The University of Hong Kong, Hong Kong SAR, China; School of Biomedical Sciences, Li Ka Shing Faculty of Medicine, The University of Hong Kong, Hong Kong SAR, China; School of Biomedical Sciences, Li Ka Shing Faculty of Medicine, The University of Hong Kong, Hong Kong SAR, China; School of Biomedical Sciences, Li Ka Shing Faculty of Medicine, The University of Hong Kong, Hong Kong SAR, China; Institute for Molecular Medicine, Ulm University, Ulm, Germany; School of Biomedical Sciences, Li Ka Shing Faculty of Medicine, The University of Hong Kong, Hong Kong SAR, China; Shenzhen Key Laboratory of Gene Regulation and Systems Biology, Department of Biology, School of Life Sciences, Southern University of Science and Technology, Shenzhen 518055, China; School of Biomedical Sciences, Li Ka Shing Faculty of Medicine, The University of Hong Kong, Hong Kong SAR, China; Shenzhen Key Laboratory of Gene Regulation and Systems Biology, Department of Biology, School of Life Sciences, Southern University of Science and Technology, Shenzhen 518055, China; STAR-UBB Institute, Babeş-Bolyai University, Cluj-Napoca, Romania; Computational Structural Biology Group, Utrecht University, The Netherlands; Max Planck Institute for Molecular Biomedicine, Münster, Germany; School of Biomedical Sciences, Li Ka Shing Faculty of Medicine, The University of Hong Kong, Hong Kong SAR, China; Centre for Translational Stem Cell Biology, Hong Kong SAR, China

## Abstract

Oct4 is essential to maintain pluripotency and has a pivotal role in establishing the germline. Its DNA-binding POU domain was recently found to bind motifs with methylated CpG elements normally associated with epigenetic silencing. However, the mode of binding and the consequences of this capability has remained unclear. Here, we show that Oct4 binds to a compact palindromic DNA element with a methylated CpG core (CpGpal) in alternative states of pluripotency and during cellular reprogramming towards induced pluripotent stem cells (iPSCs). During cellular reprogramming, typical Oct4 bound enhancers are uniformly demethylated, with the prominent exception of the CpGpal sites where DNA methylation is often maintained. We demonstrate that Oct4 cooperatively binds the CpGpal element as a homodimer, which contrasts with the ectoderm-expressed POU factor Brn2. Indeed, binding to CpGpal is Oct4-specific as other POU factors expressed in somatic cells avoid this element. Binding assays combined with structural analyses and molecular dynamic simulations show that dimeric Oct4-binding to CpGpal is driven by the POU-homeodomain whilst the POU-specific domain is detached from DNA. Collectively, we report that Oct4 exerts parts of its regulatory function in the context of methylated DNA through a DNA recognition mechanism that solely relies on its homeodomain.

## INTRODUCTION

Changes to cellular identity involve the epigenetic reprogramming of the genome including interconversions of active and inactive chromatin states. DNA binding transcription factors (TFs), chromatin remodelers, readers, writers, and erasers of epigenetic marks work together to drive these changes. The methylation of the fifth carbon of cytosines (5-methylcytosine; 5mC) at CpG dinucleotides is a critical epigenetic mark often associated with silent chromatin, particularly in somatic cells where it contributes to maintaining cell types. DNA methylation patterns are remodeled during embryonic development, germline specification and somatic cell reprogramming. These processes all require both global and locus-specific CpG methylation and demethylation (1–3). Uncovering how CpG methylation marks are read and how they affect gene regulation is critical to understanding how cellular identities are maintained and switched during cell fate reprogramming (4). Initially, CpG methylation was thought to repel TFs from their cognate binding sites which were thought to be a key reason why CpG methylation leads to repressive chromatin (5,6). However, recent evidence has cast doubt on this simple model as some TFs are attracted to methylated DNA (7–10). However, whilst TFs can bind methylated DNA it remains unclear if they utilize 5mC containing DNA as *bona fide* cis-regulatory elements, induce demethylation or drive other biological processes.

The TFs that can bind epigenetically silenced chromatin such as methylated or DNA compacted by nucleosomes to trigger biophysical and biochemical events leading to its activation are also called pioneer factors (11). The Octamer binding protein 4 (Oct4) encoded by the *Pou5f1* gene is one such pioneer TF that regulates early events of embryonic development, the maintenance of pluripotent stem cells, and the specification of the germ line (12–14). Oct4 is one component of the four-factor combination capable of converting somatic cells into induced pluripotent stem cells (iPSCs) that resemble embryonic stem cells (ESCs) derived from the inner cell mass of blastocysts (13,14). Oct4 possesses a DNA binding domain (DBD) composed of an N-terminal Pit-Oct-Unc (POU)-specific domain (POU_S_) and C-terminal POU-homeodomain (POU_HD_) connected by a flexible linker (15). This modular structure confers versatility and allows Oct4 to engage in a variety of interactions with distinct sets of DNA elements and partner factors. For example, different composite DNA elements induce Oct4 to heterodimerize with Sox2 or Sox17 in alternative dimer configurations (16,17). Palindromic binding sites (known as MORE- More palindromic Oct factor Recognition Element, and PORE- Palindromic Octamer Recognition Element,) induce topologically different Oct4 homodimers that affect co-factor recruitment in an allosteric manner (18–20). These flexible binding modes have been linked to the ability of Oct4 to directly bind and open up nucleosome core particles and its function as a pioneer factor (11,21,22). A Systematic Evolution of Ligands by EXponential enrichment (SELEX) study reported that Oct4 belongs to the group of ‘methyl-plus’ TFs that are attracted to DNA elements when their CpG sites are methylated (7). In the case of Oct4, a compact palindromic ATGCGCAT element with a CpG core (henceforth termed ‘CpGpal’) was reported to be preferentially bound by Oct4 in SELEX enriched DNA as well as in mouse ESCs. Yet, how Oct4 binds to this element and whether this binding modality contributes to its unique function in pluripotent stem cells remains unclear.

Oct4 belongs to the POU family which is a metazoan-specific TF family that evolved through the fusion of a newly evolved POU_S_ and an ancient homeodomain (23). Oct4 is part of the POU5 group of genes that are a vertebrate-specific evolutionary innovation that evolved through gene duplication from an original POU3 gene (23–25). POU3 family factors (Brn1, Brn2, Brn4 and Oct6) have prominent roles in neural specification. Indeed, Brn2 is involved in the direct lineage reprogramming of fibroblasts to induced neurons (26,27) and induces neural stem cells (28). Yet, POU3 factors are unable to replicate the ability of Oct4 to maintain and induce pluripotency (29–31). Brn2 forms dimers with Sox2 in the context of neural stem cells (NSCs) reminiscent of the Sox2/Oct4 dimer in ESCs (32). However, the dominant motif targeted by Brn2 is the MORE motif (33). Brn2 and other POU3 factors form highly cooperative homodimers on the MORE motif in contrast to Oct4 which dimerizes on the MORE with strongly reduced cooperativity (29). Re-balancing the preferences of Oct4 and POU3 factors to target SoxOct or MORE motifs through rational mutagenesis determines whether these factors reprogram somatic cells towards iPSCs (29,30). Here, we asked whether the ability of the pluripotency factor, Oct4 and the neural POU3 factor, Brn2, to differentially engage the CpGpal could contribute to their contrasting biological functions.

We performed a detailed re-analysis of genome-wide datasets combined with biochemical assays supplemented by structural modelling which showed that the modular Oct4 protein binds to the CpGpal as a homodimer with positive cooperativity. Dimeric binding is exclusively driven by the POU_HD_. Brn2 binds to the CpGpal motif with substantially diminished cooperativity and rarely targets it in cells. We report the crystal structure of Brn2 bound to MORE DNA, modeled Oct4 bound to the CpGpal, and performed molecular dynamics simulations to provide atomic insights into the dimeric binding mode. Re-analysis of Oct4 ChIP-seq data revealed that the CpGpal is constantly bound by Oct4 in particular at the onset of reprogramming and in the primed state of pluripotency. By contrast, CpGpal is barely detectable in ChIP-seq datasets of somatic POU factors. Most Oct4-bound CpGpal sites are methylated at the beginning of iPSC reprogramming and partially resist the strong genome-wide demethylation as reprogramming ensues. Collectively, we report a distinct binding configuration of Oct4 across cell fate conversions that mediate its regulatory role in the context of methylated DNA.

## MATERIALS AND METHODS

### Biological resources

B6;CBA-Tg(Pou5f1-EGFP)2Mnn/J or OG2-MEFs (RRID: IMSR_JAX:004654), LOBSTR-BL21(DE3)-RIL (Kerafast: EC1002), Rosetta™ 2 (Novagen: 71400)

### Cloning, protein expression, and purification

The POU domains, POU_HD_ and POU_S_ constructs of mouse Oct4 and Brn2 were Gateway BP cloned using primers in (Supplementary Table S1) from existing plasmids (UniProt ID, Oct4: P20263—res131–289; Brn2: P31360—res264–421) into a pENTRY vector, pDONR221 with N-terminal tobacco etch protease (TEV) cleavage site. The resulting pENTR-constructs were then cloned into either pETG20A or pETG41a expression plasmids using the GATEWAY^TM^ LR technology (Invitrogen). Constructs were transformed into Escherichia coli Rosetta™ 2 cells, grown in LB broth until OD_600_ = 0.6–0.8 before inducing with 0.5mM IPTG at 18°C for 18–22 h. For the DNA binding domain, fusion proteins were lysed by sonication (5 min total on; 4s on, 8 s off; 25% amplitude) and then purified using HisPur™ Ni-NTA Superflow Agarose (Thermo Fisher Scientific). Tags were cleaved overnight using the TEV protease followed by ion-exchange chromatography (HiTrap SP FF: Cytiva) and gel filtration (HiLoad® 16/600 Superdex® 75 pg: Cytiva) and frozen in a buffer containing (10 mM HEPES; 100 mM NaCl,10% glycerol, 0.5 mM TCEP; pH 7.0) (34). Plasmid constructs to express full-length GFP-Oct4 were a kind gift from Richard Young (35). GFP-Oct4 was expressed in LOBSTR-BL21(DE3)-RIL cells (Kerafast) and purified through HisTrap FF gradient elution (10mM-250mM Imidazole) and gel filtration (HiLoad® 16/600 Superdex® 200 pg: Cytiva).

### Electrophoretic mobility shift assay (EMSA)

#### Purified protein from bacteria

Experiments were performed as described in (34,36). Briefly, double-stranded DNA probes with 5′ Cy5 or FAM labels at one strand were prepared using an annealing buffer (20 mM Tris–HCl, 50 mM MgCl_2_, 50 mM KCl, pH 8.0). Purified protein samples and fluorescently labeled DNA were incubated for 1 h at RT in EMSA buffer. Mini gels were first pre-ran in 1× TG buffer (Biorad:1610734) at 200 V for 30 min. Then, samples were loaded, and gels were run for 30 min at 200 V at 4°C. Images are captured using an Amersham Typhoon 5 Biomolecular Imager and quantified using ImageQuantTL 7.0. DNA probes used are listed in (Supplement Table S1). Cooperativity factors for homodimers were calculated as described in (37,38) and single tube dual color EMSAs with differently labelled DNA elements were performed as described in (39).

#### Whole-cell protein extracts from human cells

HEK293T cells were cultured in DMEM (Thermo Fisher: #12100046) supplemented with 10% FBS (Gibco: #10270106) at 37°C Temp and 5% CO_2_. Cells were transfected with pLVTHM-3Xflag-Oct4 plasmids (a kind gift from the Hans R. Schöler group) (40) using a 1:2 plasmid to polyethyleneimine (w/w) ratio (Polysciences: #23966). Fresh medium was changed 16 h after transfection. 72 h post transfection, cells were dissociated with 0.05% trypsin–EDTA and washed two times with DPBS and collected by centrifugation. Cell pellets were re-suspended in lysis buffer (20 mM HEPES–KOH pH 7.8, 150 mM NaCl, 0.2 mM EDTA pH 8.0, 25% glycerol, 1 mM DTT with cOmplete™ protease inhibitor cocktail (Roche: #11836145001) added fresh). Four freeze-thaw cycles in liquid nitrogen were used to lyse the cells. Lysates were centrifuged at 14 000 × g for 10 min at 4 °C and supernatants were collected and frozen. Large (18.5 × 20 cm) 6% native PAGE gels were used for EMSAs. Protein samples and fluorescently labeled DNA were incubated for 1 h at RT. For super shift EMSA, reactions were incubated with Oct4 antibody (Santa Cruz Biotechnology: #sc-8628) for an additional 30 minutes. Gels were pre-ran at 300 V for at least 1.5 h and for another 2.5 h to separate protein/DNA complexes.

### Crystallization and structure solution

The complex was formed by mixing the Brn2 POU in [10 mM HEPES, 100 mM NaCl, pH 7.0] (4.7 mg/ml final concentration) with MORE DNA (5′-TCCTC**ATGCATATGCAT**GAGGA-3′) at a 2:1.2 molar ratio and incubated for 2 h on ice. Crystals were grown using the hanging drop vapor diffusion technique with a 2:1 ratio of complex and a buffer containing 0.2M MgCl_2_, 10% PEG4000 and 0.1M Sodium citrate tribasic dihydrate pH 5.0 at 18°C. Crystals were transferred into a cryo-solution containing 20% glycerol and 80% mother liquor and flash-frozen in liquid nitrogen. A 1.89 Å dataset was collected at BL14.1 at the BESSY II electron storage ring operated by the Helmholtz–Zentrum Berlin (41) (Supplementary Table S2). Data were indexed, integrated, and scaled using XDSapp (42). The Oct6/DNA complex (PDB-id: 2xsd) (43) was used as a search model for molecular replacement using Phaser. Refinement was performed with Phenix-Refine (44) and model building with Coot (45).

### Structural modelling

To build models of Oct4 dimer bound to the CpGpal motif, we first built with the Nucleic Acid Builder tool of Amber (www.ambermd.org) an idealized B-DNA using the sequence found in the *Nr5a2* intron: 5′-TGGATCAAGATGCTGT-**A^1^^7^T^18^**GCGC**A^23^T^24^-**ACAACTATACTGCATC-3′. By analogy with the homodimerization on the MORE motif (29), we assumed that the 2 POU_HD_ domains establish sequence-specific interactions with the AT step at position 23–24 on the strands of the Nr5a2 DNA (both numbered in 5′-3′ direction). Then we took energy-minimized models of the Oct4 homodimer bound to the MORE motif (29) and superimposed the 2 AT steps from the *Nr5a2* DNA with the corresponding AT steps from the Oct4/MORE model. To add the POU_S_ domains, we took a 2 μs classical MD simulation of apo Oct4 and superimposed the POU_HD_ domains on the corresponding POU_HD_ domains in the Oct4/MORE model. We then selected a representative Oct4 conformation that has no clashes between the POU_S_ domains and the POU_HD_ domains or the DNA. Finally, we removed the DNA and the POU_S_ domains from the Oct4/MORE model as well as the POU_HD_ domains from the apo Oct4 and puzzled together the Nr5a2 B-DNA with the domains we kept from different structures using Amber Tools. Because the two AT steps on the *Nr5a2* DNA can be read by the POU_HD_ in either direction, we build a second model in which the POU_HD_ formed sequence-specific interactions with the AT step at position 17–18 on the two strands (numbering from 5′ to 3′) which is the reversed AT step of position 23–24. We name these models ‘01’ and ‘02’, respectively. UCSF Chimera X1.1 (46) was used the inspect and analyze models and to prepare structural figures.

### Re-analysis of genome-wide datasets

A total of 16 datasets were analyzed in this study (Supplementary Table S3) (7,30,32,33,47–58).

#### ChIP- or ATAC-sequencing data processing

Sequencing data were aligned to the mm10 or hg19 genome with bowtie2 (59) using the option –very sensitive. SAM files were converted into BAM files and sorted using samtools (60). Low-quality reads and PCR duplicates were removed using sambamba (61). Correlation and relative enrichment in BAM files were visualized using the deeptools algorithms plotCorrelation and plotFingerprint (62). Peak calling was performed using macs2 (63) with a *p* value threshold of 0.0001. ENCODE blacklisted regions in mm10 were removed from peaks using bedtools (64) intersect -v. Binding intensities were normalized using MAnorm2 (65). BigWig files were generated using genomeCoverageBed and bedGraphToBigWig.

#### RNA-sequencing data processing

Aligned BAM files from RNAseq experiments of Mbd3^f/−^ TetO-OSKM MEF reprogramming towards iPSCs (52) were shared by the group of Jacob Hanna. Gene expression profiles were obtained using featureCounts (66) with GENCODE VM25 (mm10) as annotation file and GC normalized with EDAseq (67). Differential expression analysis was done using DEseq2 (68).

#### Motif discovery

Summit files from macs2 were the input for motif analysis. *De novo* motif discovery was performed using findMotifsGenome.pl in homer (47) with the options -len 12, 14, 16. Scanning for known POU motifs was performed using findMotifsGenome.pl with the option -mknown and a selected set of position weight matrices (PWMs, Supplementary Table S4). Genomic coordinates of motifs were obtained using annotatePeaks.pl (homer) with the options -m, -mbed, and -size 100, 100. For palindromic MORE and CpGpal motifs, the -norevopp option was added to avoid double counting.

#### Analysis of whole-genome bisulfite sequencing (WGBS) data

Adapter trimming of sequencing data was performed using BBduk (https://github.com/BioInfoTools/BBMap/blob/master/sh/bbduk.sh) with the options mink = 3, ktrim = r, trimq = 10, and minlength = 20. Trimmed WGBS reads were then aligned to mm10 using bismark (69) with the option -N 1. PCR duplicates were removed using deduplicate_bismark. Methylation information from deduplicated BAM files was extracted using bismark_methylation_extractor with the options –comprehensive, –no_overlap, and –no_header. Finally, methylation information of CpGs was extracted using coverage2cytosine with the option –merge_CpG. Methylation of each motif was then obtained using bedtools intersect -given (for the CpGpal) or bedtools window -w 50 using genomic motif coordinates and the output from coverage2cytosine. Only sites with 100% methylation were considered methylated.

#### Annotation of motif coordinates

Genomic coordinates of Oct4-bound motifs across all reprogramming stages were combined using bedtools merge from various datasets. Enrichment information of genomic features of each motif was obtained using Homer annotatePeaks.pl with the option -genomeOntology. Window sizes were determined according to the length of individual motifs. Distances to TSS, CpG%, and GC% were obtained from the standard output from annotatePeaks.pl. For evolutionary conservation, the mm10. 60way.phyloP60way files (70) were obtained from UCSC (http://hgdownload.cse.ucsc.edu/goldenpath/mm10/phyloP60way/mm10.60way.phyloP60way/) and converted to the glbase3 flat/hdf5 format to generate conservation plots (71). For liftover to different genomes, https://genome.ucsc.edu/cgi-bin/hgLiftOver was used. Sequence logos from fasta files were generated with Galaxy Version 3.5.0 (https://usegalaxy.org/). Datasets from (72) were used to analyze the association of motifs with epigenetic marks and chromatin-associated factors.

Additional experimental procedures for Specificity by Sequencing (Spec-seq), retrovirus production, iPSC reprogramming, and molecular dynamics simulations are available in the Supplementary Methods.

## RESULTS

### Oct4 binds CpGpal elements during pluripotency reprogramming

**Figure 1.**
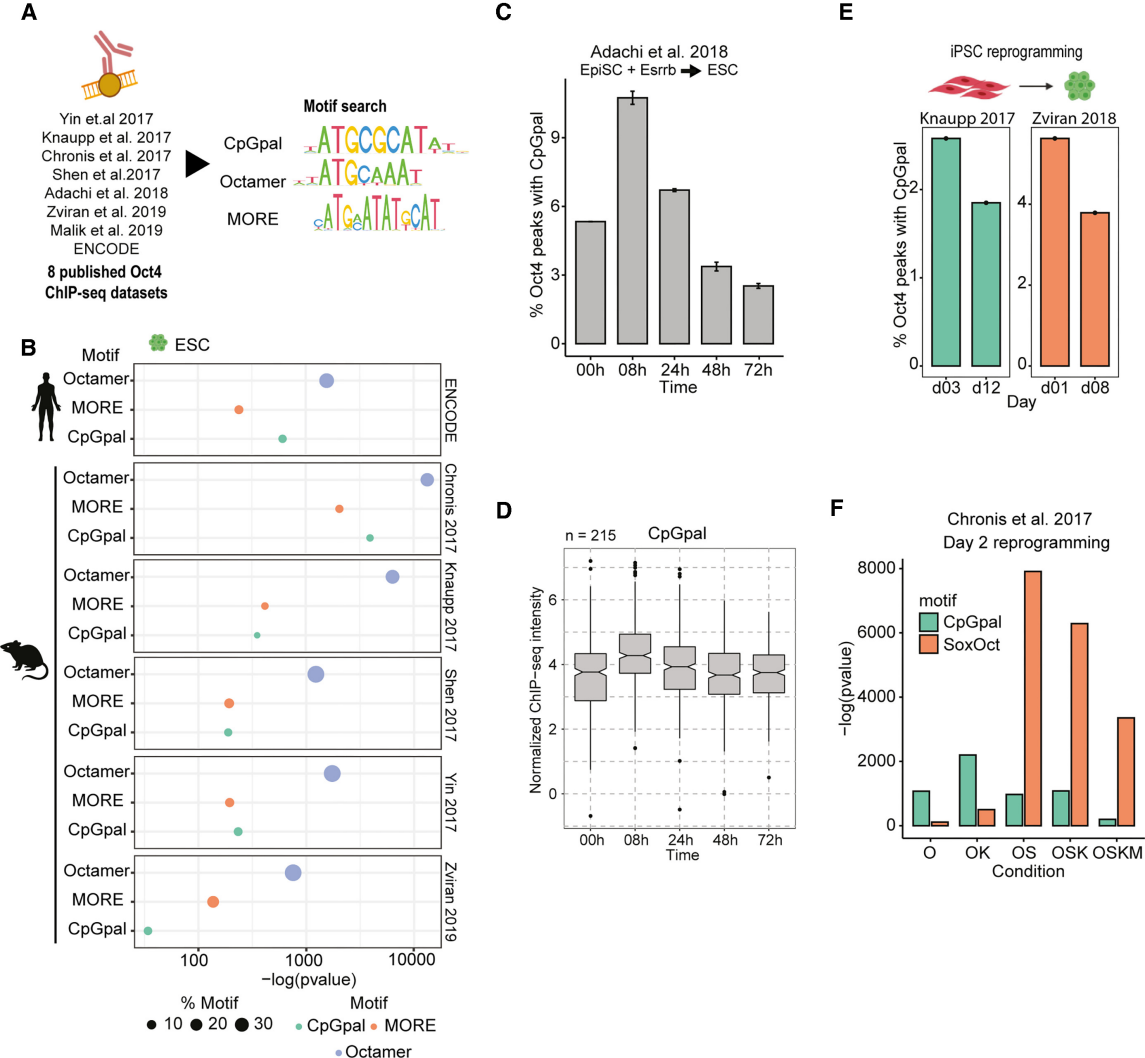
Oct4 binds the CpGpal in pluripotent cells and during reprogramming. (**A**) Schematic diagram to illustrate ChIP-seq datasets re-analyzed in this study and the sequence logos of the used motifs. (**B**) Enrichment of CpGpal, Octamer, and MORE motifs in six Oct4 ChIP-seq datasets in both human and mouse embryonic stem cells (ESCs). (**C**) Fraction of Oct4 binding sites containing a CpGpal during the reprogramming of epiblast stem cells (EpiSCs) to ESCs. (**D**) ChIP-seq peak intensities normalized by MAnorm2 of Oct4/CpGpal sites from C. (**E**) Fraction of Oct4 binding sites containing a CpGpal at time points during the reprogramming of fibroblasts to induced Pluripotent Stem Cells (iPSCs). (**F**) Bar plots of CpGpal and SoxOct motif enrichment in cells expressing indicated combinations of OSKM factors at day 2 of reprogramming. (C, E) Error bars represent standard error from ChIP-seq replicates (SEM). Abbreviations: O, Oct4; S, Sox2; K, Klf4; M, c-Myc.

To evaluate the contribution of the CpGpal motif within the cistrome of Oct4, we re-analyzed independent ChIP-seq studies performed under alternative culture conditions in mouse and human (7,30,32,33,47–54) (Supplementary Table S3) with a standardized analysis and motif discovery pipeline. We first analyzed data that lead to the initial discovery of the CpGpal motif and verified its enrichment within Oct4 sites in mouse ESCs (7). In wild-type cells, CpGpal is the second most enriched motif followed by the canonical SoxOct motif where Oct4 partners with Sox2 (Supplementary Figure S1A, Supplementary Table S5). Enrichment is further augmented in cells with a Tet1/Tet2/Tet3 triple-knock-out (TetTKO) which are hypermethylated. Conversely, in hypomethylated - Dnmt1/Dnmt3a/Dnmt3b triple knockout (DnmtTKO) ESCs occupancy of the CpGpal is strongly reduced (Supplementary Figure S1A, B). We next performed motif discovery in seven additional Oct4 ChIP-seq datasets in human or mouse ESCs as well as during mouse pluripotency reprogramming (Figure 1A) and detected enrichment of the CpGpal motif in all of them (Supplementary Table S5). The CpGpal motif was typically ranked amongst the top 3 motifs by *p* value score in ESCs and was often more enriched than the well-known MORE element (Figure 1B).

Pluripotent states can be regarded as representing a continuum mimicking early mammalian development which includes cells with variable developmental potential and altered capacity to contribute to the germline (73). *In vitro*, cells resembling the pre-implantation blastocyst exhibit naïve pluripotency with a hypomethylated genome whilst cells resembling the post-implantation blastocyst exhibit primed pluripotency with globally increased methylation levels (3,52). To evaluate whether Oct4 utilizes the CpGpal differently in these varying states of pluripotency, we analyzed Oct4 ChIP-seq data at different time points during the reprogramming of primed epiblast stem cells (EpiSCs) towards naïve ESCs (51). Interestingly, the CpGpal motifs were preferentially targeted in the primed pluripotency state and at the onset of the conversion to a naïve state (Figure 1C-D). The reprogramming of somatic cell fibroblasts towards iPSCs is accompanied by global DNA demethylation (48,52). We wondered whether the reprogramming factor Oct4 occupies the CpGpal motif during iPSC reprogramming. We found that Oct4 binds CpGpal motifs in particular at the onset of reprogramming (Figure 1E). A study by the Plath laboratory evaluated the binding landscape of different reprogramming factor combinations on day two of pluripotency reprogramming (50). When Oct4 was expressed alone, the fraction of peaks with the CpGpal motif is the highest (Supplementary Figure S1C). The presence of Sox2 leads to a notable reduction in CpGpal binding with concomitant increased occupancy of SoxOct motifs (Figure 1F). This demonstrates that co-factors impact the relative preferences for regulatory DNA. In sum, the CpGpal makes a significant contribution to the binding landscape of Oct4 and is occupied during the reprogramming of somatic and primed cells to pluripotency.

### CpGpal sites are epigenetically repressed at the onset of reprogramming and partially resist demethylation

**Figure 2.**
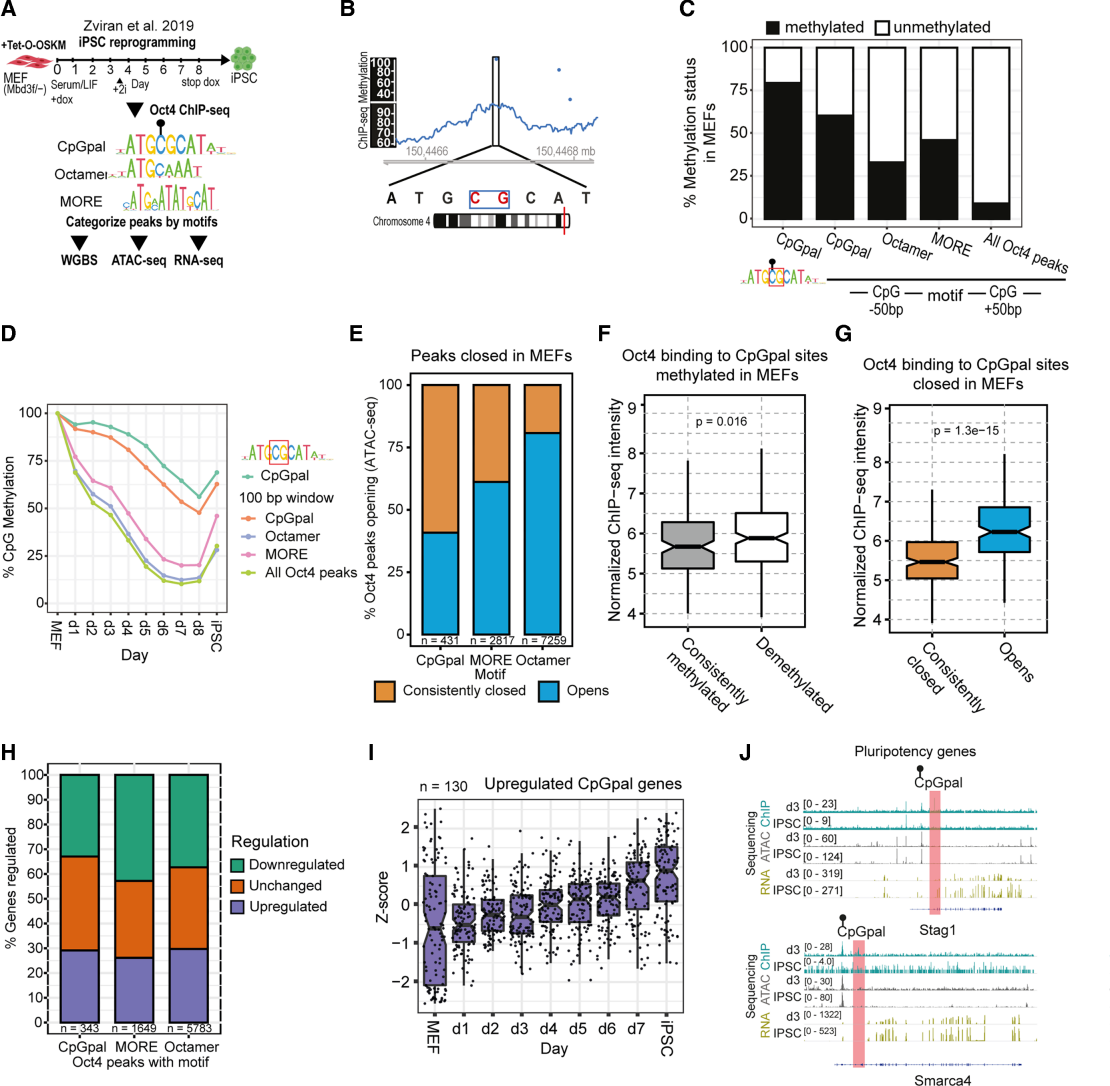
CpGpal sites are methylated in fibroblasts and often maintain methylation during iPSC reprogramming. (**A**) Schematic diagram of the iPSC reprogramming system providing the datasets for our re-analysis (52). Oct4 peaks were categorized by the presence of CpGpal, MORE or Octamer motifs and these peak sets were compared for context dependent methylation, chromatin accessibility and gene expression. (**B**) Example of an Oct4 bound CpGpal element that is methylated in MEFs. (**C**) The methylation status in MEFs for indicated categories of Oct4 binding sites. Methylation was measured for the central CpG of the CpGpal and using a 100 bp window centered on the motifs. (**D**) Methylation dynamics throughout iPSC reprogramming for the subset of Oct4 peaks in C that are methylated in MEFs. (**E**) The fraction of Oct4 binding sites that are closed in MEFs and open up or remain closed are compared for the indicated motifs. (**F**) A comparison of Oct4 ChIP-seq binding intensities to CpGpal sites that remain methylated or get demethylated with respect to MEFs. (**G**) A comparison of Oct4 ChIP-seq binding intensities to CpGpal sites that remain closed or open up during iPSC reprogramming. (**H**) Genes were linked to indicated Oct4 binding site categories and the differential expression was compared between days 1 and 7 of iPSC reprogramming. (**I**) Expression 130 genes associated with Oct4 bound CpGpal throughout iPSC reprogramming. (**J**) Two examples of upregulated pluripotency genes containing an intronic methylated CpGpal site that is bound by Oct4. (F, G) *p*values were determined by the Wilcoxon rank sum test. Boxes represent the interquartile range with a median line. Whiskers indicate last values within 1.5 times the interquartile range. Notches display 95% confidence interval. Abbreviations: MEFs, mouse embryonic fibroblasts; iPSC, induced pluripotent stem cells.

We next focused on data from the Jacob Hanna group as they provide a high-resolution map of Oct4 binding on each day of reprogramming alongside measurements of gene expression, chromatin openness, and DNA methylation (52) (Figure 2A). We detected 439 instances of CpGpal sites within Oct4 peaks in these datasets. During reprogramming to pluripotency, the genomes of somatic cells undergo gradual and widespread DNA demethylation (52,74). To evaluate the methylation dynamics of loci targeted by Oct4, we re-analyzed whole-genome bisulfite sequencing (WGBS) data that provide methylation information at single-nucleotide resolution (Figure 2B). We inspected the methylation status of the CpG dinucleotide at the core of the ATGCpGCAT motif in MEFs and the average methylation status within a 100-bp window flanking CpGpal, Octamer, MORE, and all Oct4 binding sites (Figure 2C, D). More than 75% of CpGpal sites bound by Oct4 are methylated in MEFs (Figure 2C). Most of the methylated binding loci of Oct4 gradually lose methylation during reprogramming and are largely unmethylated by day 8 (Figure 2D), which follows the pattern of genome-wide DNA demethylation (52). Yet, more than half of the Oct4/CpGpal sites remain methylated at day 8 (Figure 2D). We next looked at the relationship between CpG methylation and chromatin accessibility, as CpG methylation is often associated with closed chromatin (1–3). Most sites bound by Oct4 are closed in MEFs and open up during reprogramming (Supplementary Figure S2A, Figure 2E). However, whilst Oct4/MORE and Oct4/Octamer predominantly open up, more than half of the Oct4/CpGpal sites remain closed during reprogramming (Figure 2E). The Oct4 ChIP-seq signal at CpGpal sites is of similar strength irrespective as to whether sites remain methylated or become demethylated during the reprogramming of MEFs towards iPSCs showing the methylation does not impede binding by Oct4 (Figure 2F). Yet, Oct4/CpGpal sites that open up compared to MEFs have significantly higher ChIP-seq signals than those that remain closed, suggesting a link between binding strength and pioneer activity (Figure 2G).

We next compared the expression of genes associated with Oct4 bound motifs (CpGpal, MORE, Octamer) at day 1 versus day 7 of iPSC reprogramming. The fraction of upregulated genes is similar for all three peak categories (Figure 2H). Oct4/CpGpal-associated genes that are upregulated during reprogramming, include several known pluripotency genes (Figure 2I, J, Supplementary Figure S2B). Although Oct4 binding to CpGpal sites is less likely to lead to demethylation or chromatin opening, Oct4/CpGpal sites have a similar effect on nearby genes as the canonical Oct4/Octamer or Oct4/MORE configurations. Examples are *Stag1* and *Smarca4* which remain methylated and are upregulated during reprogramming (Figure 2J). The pioneer model suggests chromatin opening and demethylation precede gene activation. This applies to the majority of Oct4 binding sites. Yet, there might be a subset of CpGpal sites where Oct4 exerts its regulatory role despite signatures of silent chromatin.

### CpGpal sites show reduced demethylation and elevated *de novo* methylation in ESCs

To further examine the methylation dynamics of CpGpal sites, we inspected the rate of *de novo* methylation catalyzed by Dnmt3a/b or passive demethylation from reduced Dnmt1 maintenance methylation. These rates were measured in an elegant study that provides genome-wide data for methylation turnover at nearly 1 million CpG sites in mouse ESCs (75). For this analysis, we first collected a superset of Oct4-bound CpGpal loci from three independent Oct4 ChIP-seq studies (Supplementary Figure S2C). Intersecting these Oct4 binding sites with CpG coordinates and methylation turnover data showed that CpGpal sites have reduced rates of passive demethylation, and accelerated rates of *de novo* methylation compared to other categories of Oct4 binding sites (Supplementary Figure S2D, E). Consistently, CpGpal sites show elevated levels of methylation (5mC), hydroxy-methylation (5hmC), and Dnmt3a/b (Supplementary Figure S2F-H). These observations together with the finding that Oct4 bound CpGpal sites remain largely methylated during pluripotency reprogramming (Figure 2C, D) leads us to infer that Oct4 is not actively involved in the erasure of 5mC methylation within the context of CpGpal sites.

### CpGpal sites undergo hereditary C to T transitions

**Figure 3.**
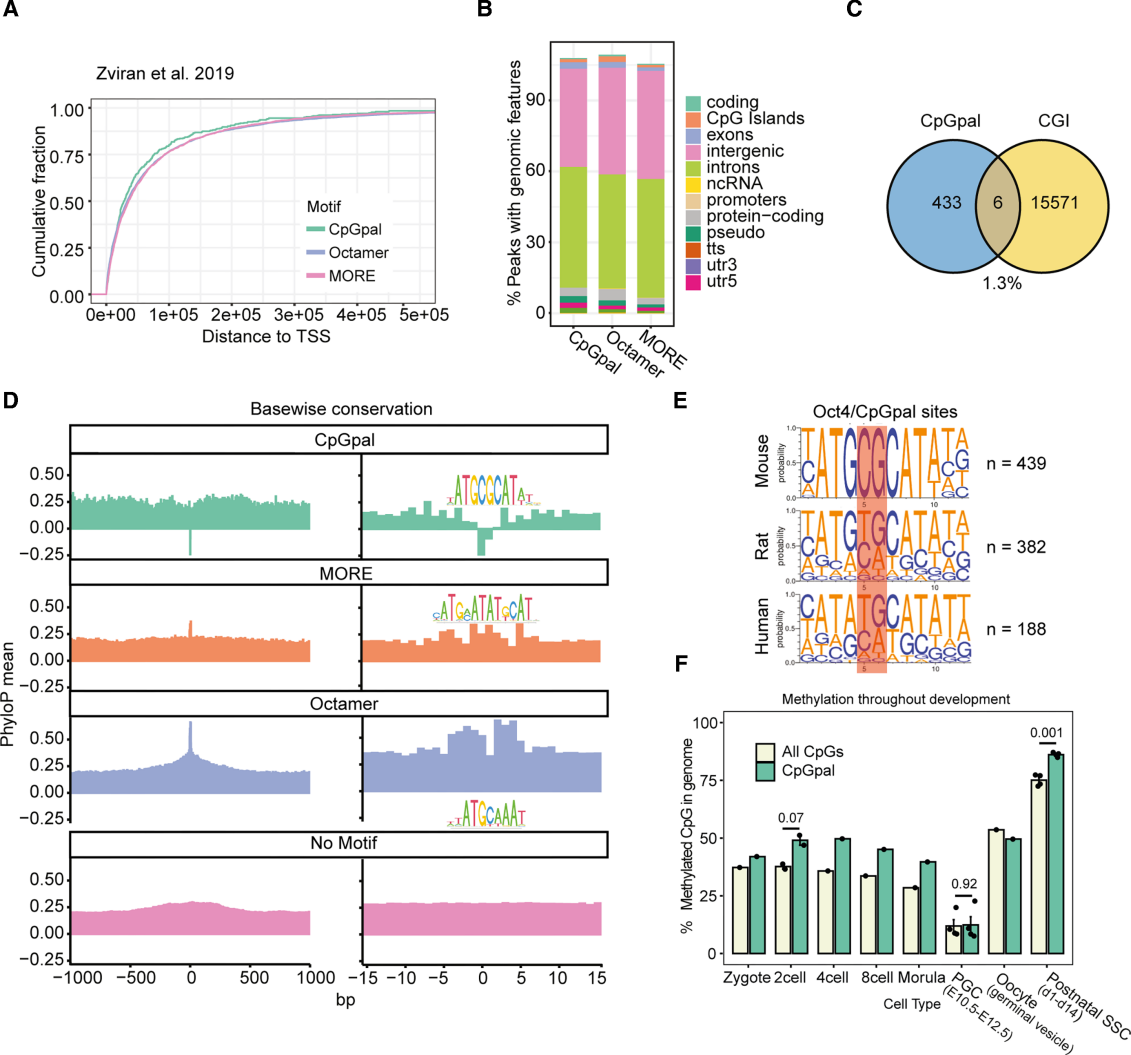
Annotation and conservation of CpGpal sites. (**A**) Cumulative distance of Oct4 binding sites categorized by the presence of indicated motifs to transcription start sites (TSS). (**B**) Genomic annotations associated with Oct4 binding site categories (CpGpal, Octamer, MORE). (**C**) The intersection of Oct4 bound CpGpal sites and CpG islands (CGIs) show that they rarely coincide. (**D**) Evolutionary conservation of Oct4 binding site locations with matches to indicated motifs using the Placental Mammal Basewise Conservation by PhyloP (phyloP60way) track from multiple alignments of 60 vertebrate species. (**E**) Sequence logos of mouse Oct4/CpGpal sites and logos using sequences at equivalent positions after lifting over the mouse coordinates to rat (rn6) and human (hg38) genomes. (**F**) Bar plots showing the fractional methylation in indicated cell types for all CpGs in the mouse genome and for the subset of CpG’s within CpGpal sites (55–58). (F) For *n* > 2, data are shown as mean ± SEM and *p*values were determined from an unpaired Student's *t* test. Abbreviations: PGC, primordial germ cells; SSC, spermatogonial stem cells; E, embryonic day, d, postnatal day.

To investigate whether CpGpal elements are linked to specific genomic features, we probed its distance to transcription start sites (TSS) and annotated sites for genomic elements. All three binding sites show comparable TSS distances and are often located within introns (Figure 3A, B). Importantly, CpGpal sites are not enriched in CpG islands that often serve as promoters of housekeeping genes (76) (Figure 3C). TF binding sites can either be evolutionarily conserved or evolve rapidly through transposition events or sequence substitutions (77). To examine the conservation at Oct4 bound motif sites, we used phyloP evolutionary conservation scores. Sites with Octamer or MORE motifs show evident evolutionary conservation at the center of the motif site which peaks at nucleotide positions constrained within the PWM (Figure 3D). In contrast, CpGpal sites are not conserved. Rather, they show negative phyloP scores at the central CpG dinucleotide subject to methylation suggesting accelerated evolution. C to T transitions are common somatic and germline mutations (78). These substitutions are a direct consequence of the oxidative deamination of methylated cytosines. Therefore, cytosine methylation makes this nucleotide particularly vulnerable to point mutations (79). To further examine the conservation of the 439 mouse CpGpal sites bound by Oct4, we retrieved corresponding sequences from rat or human genomes. We recovered 382 rat and 188 human sequences. Next, we generated sequence logos to examine which nucleotides changed in these species. We found that the ATG flanks remain conserved but the central CpG is often converted into TA dinucleotides, suggesting frequent C-T transitions (Figure 3E). The genome goes through demethylation and re-methylation waves during development in a sex-specific manner (80) (Supplementary Figure S3). We next examined the methylation status of CpGpal sites in mouse embryonic development and germ cell specification (55–58). We find that throughout development, CpGpal is indeed highly methylated and at times exceeds global CpG methylation levels (Figure 3F). CpGpal is also methylated in primordial germ cells (PGCs) as well as both male (post-natal spermatogonial stem cells) and female (oocytes) germ cells. Oct4 is abundantly expressed at the 8-cell stage, in pluripotent cells and the germ line (81,82), where it is critical in the development of PGCs (12). We speculate that binding to these methylated sites contributes to germ-cell development.

### Oct4 cooperatively homodimerizes on the CpGpal motif

**Figure 4.**
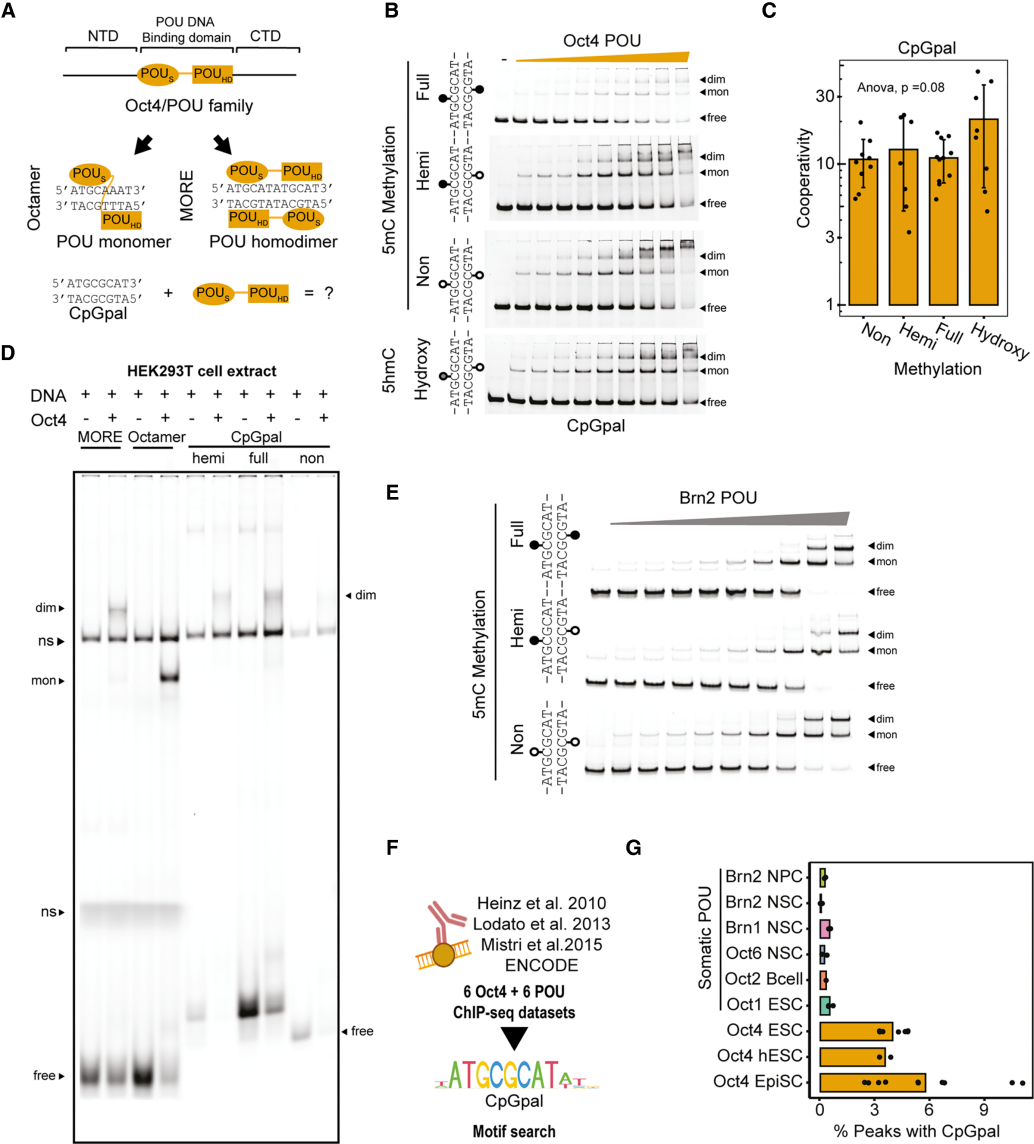
Oct4 forms specific homodimers on the CpGpal element. (**A**) Schema of the domain structure of Oct4 (top) and monomeric and dimeric binding modes of Oct4 to Octamer or MORE (bottom). (**B**) Representative EMSA titrations from 2–3 replicate experiments with 50 nM Cy5-labeled CpGpal DNA probes and increasing concentrations of the Oct4 POU (25–800 nM). (**C**) Calculation of cooperativity factors (ω) for EMSAs in (B) according to the formula in S4C (*n* = 7–10). (**D**) Representative EMSA from two replicate experiments using full-length Oct4 protein from HEK293T whole cell extracts and indicated DNA probes of Octamer, MORE and methylation states of the CpGpal. (**E**) Representative EMSA titrations from two replicate experiments with 50 nM Cy5 labeled CpGpal DNA probes and increasing concentrations of the Brn2 POU (10–500 nM). (**F**) ChIP-seq datasets of somatic POU factors re-analyzed in this study. (**G**) Percentage of ChIP-seq binding sites of the indicated POU factors with matches to the CpGpal motif. (B, E) Arrows denote free DNA (free) or shifted probes where Oct4 binds as monomer (mon) or dimer (dim). (C) Data are shown as mean ± standard deviation (sd). *p*value was determined from a one-way ANOVA. Cooperativity factors (ω) in (C) were calculated for lanes where the fractional contribution of each of the three bands was at least 0.05.

To study the association of Oct4 with the CpGpal element *in vitro*, we purified the POU domain of Oct4 to homogeneity and verified its ability to bind Octamer DNA with nanomolar affinity (Figure 4A, Supplementary Figure S4A, B). Next, we performed EMSAs using DNA probes with methylated and unmethylated CpGpal DNA (Figure 4B). We found that Oct4 forms homodimers on these elements with positive cooperativity using a previously established model to determine the cooperativity factor (ω) for homodimeric binding to palindromic DNA at equilibrium (37) (Figure 4C, Supplementary Figure S4C). The 5mC or 5hmC marks had no effect on homodimerization (Figure 4C). To further examine the new binding modality of Oct4, we re-analyzed data from a specificity by sequencing (Spec-seq) experiment (34) that included a sequence library for octameric DNA where the four central nucleotides are randomized (ATNNNNAT) (Supplementary Figure S4D). We noted a weak band which we excluded from our previous analysis as we were interested to probe the specificity for monomeric binding to Octamer DNA (34). Indeed, amongst the 256 sequences, the ATGCGCAT element corresponding to the CpGpal has the highest fractional enrichment within the dimer band (Supplementary Figure S4E). We also performed Spec-seq with an ATGNNNAT library that was enzymatically methylated with the CpG Methyltransferase—M.SssI. The analysis revealed that Oct4 retains its specificity for cognate binding elements following methylation (Supplementary Figure S4F) and binding energies for methylated and unmethylated DNA elements are similar (Supplementary Figure S4G).

We next examined the thermal unfolding of CpGpal/Oct4 complexes vis-à-vis Octamer/Oct4 complexes using differential scanning fluorometry (DSF). Oct4 is stabilized by the presence of both DNA elements with indistinguishable unfolding curves, suggesting equally tight binding (Supplementary Figure S4H). Lastly, we examined Oct4/CpGpal complex formation using full-length proteins. First, we verified dimeric binding using full-length GFP-Oct4 purified from bacteria (Supplementary Figure S4I). Next, we performed a modified EMSA assay based on HEK293T cell extracts expressing Oct4. Oct4 was able to form dimers on CpGpal demonstrating that the translation and processing in human cells as well as the presence of N-and C-terminal domains do not change its mode of binding (Figure 4D, Supplementary Figure S4J, K). In sum, we find that Oct4 binds the short palindromic CpGpal as a dimer with positive cooperativity.

### Brn2 dimerizes poorly on the CpGpal and evades it in neural stem cells

Oct4 is a unique POU factor critical for reprogramming and pluripotency as it cannot be substituted for by other POU family members (30,83,84). To examine whether the association with methylated DNA contributes to its uniqueness, we tested whether other POU family proteins can bind to the CpGpal *in vitro*. We choose the POU3 family protein Brn2 for this analysis for its prominent roles in neural stem cells and its potency in neural reprogramming (28,85–87). We purified the POU domain of Brn2 and verified its nanomolar affinity for Octamer DNA (Supplementary Figure S5A). EMSAs show that Brn2 can also bind to CpGpal but dimer formation is strongly reduced compared to Oct4. Dimer bands are only seen when the free DNA is depleted while monomeric bands predominate at equilibrium conditions. This is indicative of a binding mode with negative cooperativity (Figure 4E). Brn2 is known to homodimerize with higher cooperativity than Oct4 on MORE DNA (29). To further evaluate differences between Oct4 and Brn2, we exposed these proteins simultaneously with FAM-labelled CpGpal and Cy5-labeled MORE DNA in single tube EMSA reactions (Supplementary Figure S5B, C). The fluorometric comparison of dimer fractions confirms a strong preference of Brn2 for the MORE whilst Oct4 more effectively dimerizes on the CpGpal (Supplementary Figure S5D).

This raised the question as to whether the CpGpal is bound by Brn2 and other somatic POU factors in cellular chromatin. To test this, we re-processed ChIP-seq datasets for six somatic POU factors (Figure 4F). We observed POU motifs such as the Octamer or MORE motif as the top *de novo* motifs in these datasets. However, the CpGpal motif was not among the top motifs (Supplementary Table S6). Motif scanning showed that <0.5% of sites contained matches to the CpGpal. This is in stark contrast to Oct4 datasets showing matches within 3–10% of sites with highly significant enrichment scores (Figure 4G, Supplementary Table S5). Collectively, biochemical assays combined with a re-analysis of genome-wide binding data reveal that the dimeric binding to the CpGpal is a unique feature of Oct4 which sets it apart from Brn2 and other members of the POU family.

### The homeodomain of Oct4 is necessary and sufficient to bind the CpGpal

**Figure 5.**
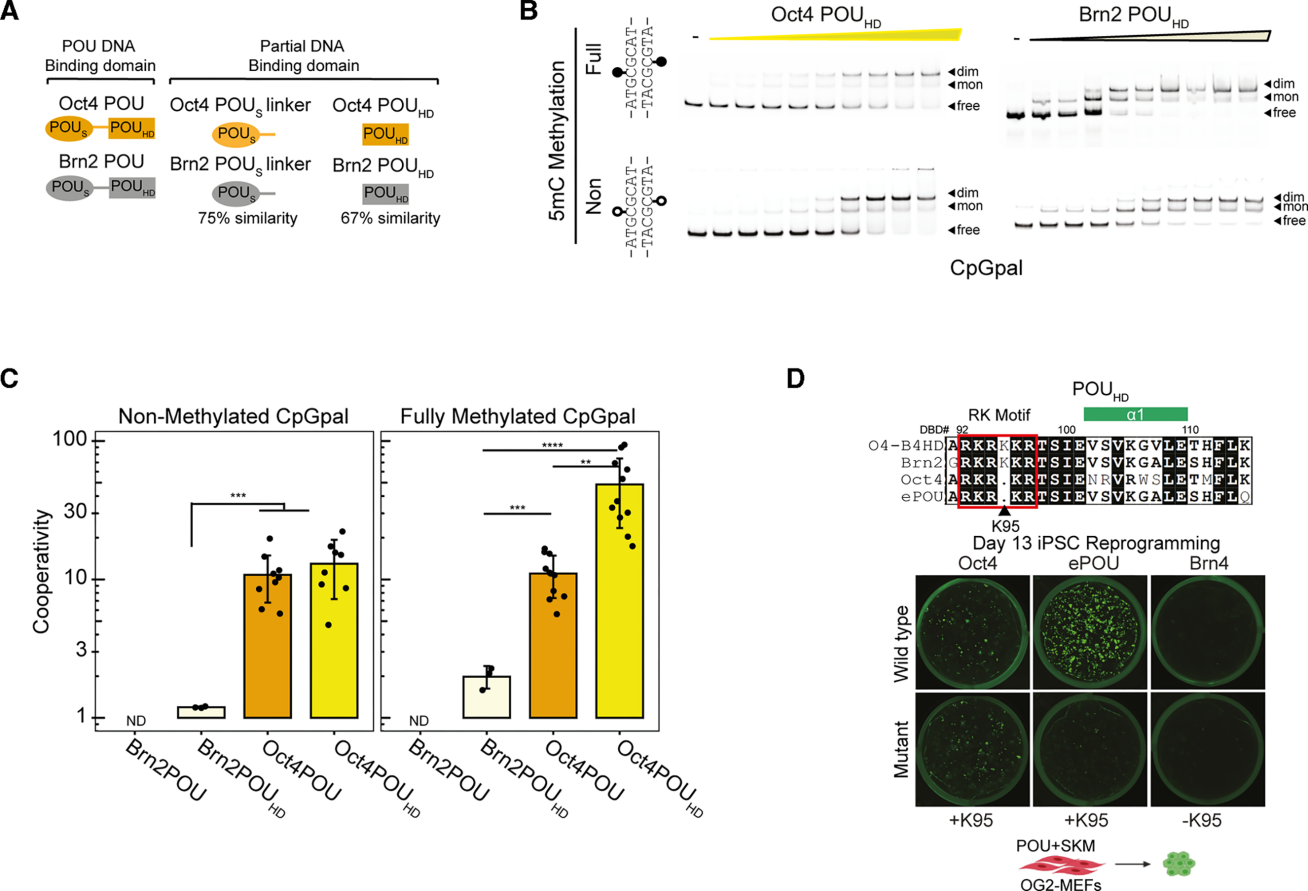
The homeodomain of Oct4 is necessary and sufficient for cooperative dimerization on the CpGpal. (**A**) Schema of the Oct4 and Brn2 protein truncation constructs designed for binding experiments.(**B**) Representative EMSA titrations from 2–3 replicate experiments comparing the binding of the POU_HD_ sub-domain of Oct4 and Brn2 proteins with 50 nM Cy5 labeled fully and non-methylated CpGpal probes. (**C**) Cooperativity factors (ω) for EMSAs of POU_HD_ sub-domain in (B) plotted along with data points of Oct4 POU in Figure 4C and Brn2 POU in Figure 4E for fully and non-methylated CpGpal (*n* = 3–10). (**D**) Multiple sequence alignment of the POU_HD_ N-terminal tail containing the RK motif (top, POU domain numbering). Mouse retroviral iPSC reprogramming using Oct4, ePOU and Brn4 in comparision to K95 insertion (Oct4/ePOU) or K95 deletion (Brn4) mutants SKM (*n* = 2). Whole-well scans of 12 well plates at day 13 (bottom). (B) Arrows are highlighting free DNA (free), monomer (mon) or dimer (dim) bound DNA bands. (C) Data are shown as mean ± sd and ND means Not Determined as for the highly competitive binding no condition can be found where the fraction of each of the three bands per lane contributes at least 0.05. Adjusted *p* values were determined from Tukey's (honestly significant difference) test after a one-way ANOVA. Cooperativity factors (ω) in (C) were calculated according to Supplementary Figure S4C and (37). Abbreviations: S,S ox2; K,K lf4; M, c-Myc.

To test which sub-domain mediates Oct4 dimerization on the CpGpal, we designed truncation constructs of the individual POU_S_ and POU_HD_ of Oct4 as well as of Brn2 (Figure 5A, Supplementary Figure S6A, Supplementary Table S7). The homeodomain constructs could be purified with good yield and high purity. However, our attempts to purify the POU_S_ domain of Oct4 failed although we expressed multiple constructs with alternative domain boundaries and attempted refolding from inclusion bodies. Yet, the POU_S_ of Brn2 could be purified and was used instead of the Oct4 POU_S_ to evaluate the binding of the subdomains (Supplementary Figure S6A). Both, the Oct4 and Brn2 POU_HD_ bind the Octamer DNA with high affinity (Supplementary Figure S6B, C). Interestingly, the Oct4 POU_HD_ could homodimerize on Octamer DNA (Supplementary Figure S6B). This dimerization is not detectable for the full POU domains of both proteins and the POU_HD_ of Brn2. Presumably, for its negative net charge (Supplementary Figure S6D), the isolated POU_S_ domain of Brn2 did not show DNA binding to Octamer DNA (Supplementary Figure S6C). This is consistent with previous studies demonstrating that the POU_S_ of Oct1 only binds Octamer DNA at high concentrations with strict sequence constraints (88,89).

We next probed the binding of the isolated POU_HD_ to the CpGpal element. We found that the Oct4 POU_HD_ homodimerizes even more efficiently than the bipartite Oct4 POU domain on fully methylated CpGpal DNA (Figure 5B, C). The Brn2 POU_HD_ can also bind and dimerize on the CpGpal motif but with strongly reduced cooperativity (Figure 5C). The methylation status had only a minor effect on the dimerization of the Oct4 POU_HD_ (Figure 5C). We further verified the results using a CpGpal element present in genomic DNA near the *Zfp957* gene (Supplementary Figure S6E, F). In conclusion, the POU_HD_ mediates the cooperative dimerization of Oct4 on the CpGpal without the help of the POU_S_. This is in stark contrast to previously reported homodimers on MORE or PORE elements that require both subdomains.

### A variable residue within the N-terminal tail of the homeodomain impacts pluripotency reprogramming

The unique cooperative dimerization of Oct4 POU_HD_ on CpGpal inspired us to probe into the sequence differences between Oct4 and Brn2 POU_HD_ (Figure 5D). Amino acids involved in sequence specific base readout are conserved within the POU family. Yet, there are differences within the N-terminal arm of the homeodomain. The N-terminal arm is enriched for basic amino acids and serves as a nuclear localization signal (90). This motif contains a stretch of basic RKRKR (Oct4) or RKRKKR (POU3) residues (Figure 5D, hence also known as RK motif). The removal of this region is deleterious to DNA and nucleosome binding (91). Likewise, alanine mutants of the individual residues in the RK motif impaired DNA binding, transactivation, and reprogramming (92). We recently reported an artificially evolved and enhanced POU factor (ePOU) that outperforms wild-type Oct4 in pluripotency reprogramming (34). In the ePOU, a part of the Oct4 POU_HD_ was replaced by the Brn2 POU_HD_. The gain-of-function effect of this domain swap was surprising as in another study the replacement of the Oct4 POU_HD_ with that of Brn4 POU_HD_ (O4-B4HD) led to a reduction of iPSC reprogramming activity (31). We inspected the sequences and noted that O4-B4HD contains an additional lysine within the region of the RK motif (Figure 5D). The additional residue is present in most POU family members except within the POU4 and POU5 groups (90). We reasoned that the difference in the RK motif might explain the functional differences between ePOU and O4-B4HD. Indeed, when we inserted an additional K into ePOU, its ability to generate iPSCs was strongly reduced (Supplementary Figure S6G, H). This underscores the functional relevance of the RK motif of the POU_HD_ and demonstrates that subtle alterations within its sequence have profound consequences in pluripotency reprogramming.

### Structural basis for the binding of Brn2 to MORE DNA

**Figure 6.**
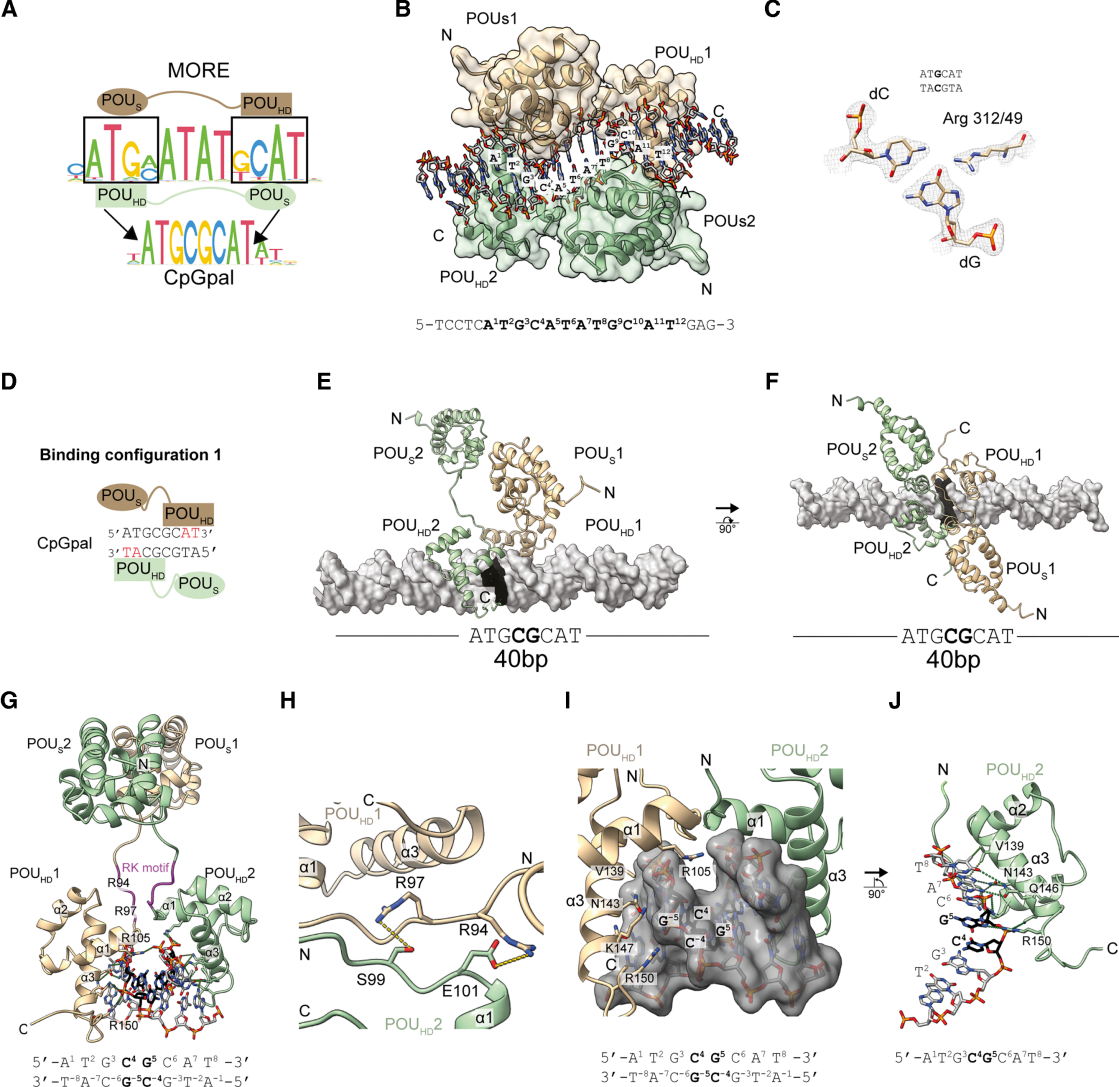
Structural basis for the Oct4 dimerization driven by the homeodomain. (**A**) Schematic how the POU_S_ and POU_HD_ bind to the MORE and implications for CpGpal binding. (**B**) 1.9 Å crystal structure of the Brn2 POU homodimer bound to the MORE DNA (PDB: 7XRC). (**C**) 2Fo – Fc electron density contoured at the 2σ level around Arg312 of full length Brn2 (Arg49 in DBD numbering) and the GC base pair it interacts with. (**D**) Cartoon of a Oct4 POU bound to CpGpal where the POU_HD_ binds the AT steps at the flanks (red) using residues V139, N143 and Q146 (POU domain numbering) in an orientation as observed in the Brn2-MORE DNA complex. (**E**, **F**) Energy minimized structural models of Oct4 POU bound to CpGpal in configuration 1 where the POU_HD_ drives dimer whilst the POU_S_ is detached from the DNA. (**G**) Focused view onto the octameric CpGpal bound by Oct4 in configuration 1. In this model, the RK motif is engaged in protein–protein interaction, R105 targets the minor groove of the central CpG and R150 binds the G of the CpG via the major groove. (**H**) Amino acids within the RK motif of two POU_HD_ molecules may facilitate dimerization (R95, R97, S99, E101). Orientation was changed with respect to (G). **(I, J)** Enlarged views of protein–DNA contacts in the same orientation as in (G). Residues of the recognition helix α3 that bind the AT flanks (V139, N143, Q146) and residues that contact the core CpG of the CpGpal (R105, K147, R150) are shown as sticks and marked for separate monomers of POU_HD_1(I) and POU_HD_2(J). The DNA is shown as a surface in (I) to highlight the insertion of R105 into the minor groove. In (J), POU_HD_1 and one of the DNA strands are omitted for a clearer view onto the major groove contact of R150 with G_5_. (D–J) Models show human Oct4 POU domains consisting of POU_S_ and POU_HD_ of two molecules (colored tan (molecule 1) sea green (molecule 2)). Models are made with a 40 bp genomic DNA (grey) sequence from the totipotency gene *Nr5a2* (121) locus containing the CpGpal motif at the center. Proteins are shown as ribbons and the DNA as surfaces or sticks with the CpG core of the motif highlighted in black. (G–J). Highlighted residues follow the POU DBD numbering convention. N/C termini and helices of the POU_HD_ are labelled in some panels.

The CpGpal element shares similar features with the palindromic MORE element. It retains the four base pair flanks but lacks the central ATAT stretch (ATGCATATGCAT vs ATGCGCAT) (Figure 6A). Given the similarity between MORE and CpGpal, we wondered whether they co-occur within the cistrome of Oct4. Yet, less than 1% of MORE sites directly intersect with CpGpal coordinates, and only 1.2% co-occur within a 200 bp window (Supplementary Figure S7A). This demonstrates that Oct4/CpGpal and Oct4/MORE represent distinct binding sites that rarely overlap. POU3 factors such as Oct6 and Brn2 dimerize on the MORE with more positive cooperativity compared to Oct4 as they encode non-polar amino acids such as methionine at position 151 of the POU domain contrary to the polar serine of Oct4 (29). Switching this amino acid enhances the dimerization of Oct4S151M on the MORE and reduces the MORE dimerization of Oct6M151S. In Oct4S151M ChIP-seq peaks the MORE motif is more strongly enriched compared to wild-type Oct4 (30). By contrast, the enrichment of CpGpal motifs is similar for wild-type Oct4 and Oct4S151M (Supplementary Figure S7B). This shows that dimerization-enhancing mutations on the MORE do not increase binding to the CpGpal and indicates that the mechanism for dimeric binding is fundamentally different.

To reveal the structural basis for Brn2 dimerization on MORE, we performed crystallization trials. We could generate crystals of Brn2 bound to MORE DNA diffracting to 1.9 Å resolution (Figure 6B, C, Supplementary Figure S7C, Supplementary Table S2). The Brn2/MORE structure resembles previously reported Oct1 and Oct6 structures (43,93) with POU_S_ and POU_HD_ domains positioned at opposite faces of the DNA arranged around a two-fold crystallographic axis (Figure 6A, B). The POU_HD_ and POU_S_ engage in direct intermolecular protein-protein contacts mediated by hydrophobic residues (POU_S_-S6, L9, L53, F57, M60 with POU_HD_ 151M; POU_S_ L53 with POU_HD_ T152) (Supplementary Figure S7D).

### Basis for Oct4 dimerization on the CpGpal

To gain structural insight into the dimerization of Oct4 on CpGpal, we generated various Oct4 or Brn2 constructs and used DNA elements with different overhangs. Despite intense efforts, we were unable to obtain diffracting crystals of POU/CpGpal complexes. Yet, the Brn2/MORE structure presented here along with various previously reported POU/DNA complexes (20,43,93,94) have provided detailed insights which amino acids mediate specific DNA contacts. These experimental structures served as guides to construct structural models as to how Oct4 binds the CpGpal (Figure 6D–F, Supplementary Figure S7E). On MORE, the 5′-ATGC site is bound by POU_S_ and the reverse complement 3′-TACG by the POU_HD_ (Figure 6A, B). As the amino acids mediating the binding to these half sites are highly conserved within the POU family, we predict an analogous binding mode to the CpGpal and eliminated the central ATAT from MORE to turn it into a CpGpal element. Because of this, two ATGC-bound POU_S_ molecules are subject to severe clashes, suggesting that they cannot co-exist on the 8 base-pair CpGpal element (Supplementary Figure S7E). By contrast, a model of two POU_HD_ domains binding the 5′-GCAT as seen on the MORE does not show any clashes (Figure 6D–F). Consistently, two POU_HD_ alone effectively dimerize on the CpGpal but the isolated POU_S_ of Brn2 does not (Figure 5B, C). The conserved residues V139, N143, and Q146 from the POU_HD_ recognition helix α3 form specific contacts with the AT steps at the flanks of the CpGpal (ATGCpGCAT) (Figure 6G–J). In support of this model, mutations of the AT residues affect overall binding and dimer formation (Supplementary Figure S7F). As the AT steps can be read in either direction, we present another hypothetical configuration 2 where the POU_HD_ is binding the AT of the opposite strand (Supplementary Figure S8A–E). We equilibrated models of both configurations and performed 470 ns molecular dynamics (MD) simulation (Supplementary Movies S1 and S2). MD simulations for both binding configurations are stable providing further evidence that our models are plausible (95) (Supplementary Figure S8F). We consider configuration 1, where the POU_HD_ binds the GCAT half-site reminiscent of the MORE, to be the more likely binding mode for several reasons. First, in this configuration, the basic RK motif of the POU_HD_ including the variable K95 that affects reprogramming are engaged in intermolecular interactions (Figure 6G, H). The N-terminal tail (containing the RK motif) and linker are structurally flexible, often disordered in reported structures, and can undergo conformational adjustments to accommodate alternative binding partners (Supplementary Figure S8G, H). Second, R105 of helix α1 binds the phosphates of the minor groove side of the CpG step (Figure 6G, I). CpG methylation has been predicted to narrow minor groove width (MGW) (96,97). Narrowed MGW increases the electrostatic potential which can be specifically recognized by arginines (98). Thus, R105 might be involved in an indirect readout mechanism of DNA shape (97,99). Third, R150, located C-terminally of the recognition helix α3, is approaching the G^5^ of the central C^4^pG^5^ step suggesting a direct readout of this base (Figure 6J). However, we do not observe direct contact of the C^4^ subject to methylation (Figure 6I, J). This is consistent with our binding assays which suggested that dimerization proceeds similarly on methylated and unmethylated DNA. This binding mode differs from the methylation-sensitive CpG reader, HOXB13, that directly binds the methylated cytosine with hydrophobic residues of its recognition helix α3 (7) (Supplementary Figure S8I, J). In contrast to binding configuration 1, configuration 2 does not show any intermolecular protein-protein interactions and lacks direct base contacts with the C^4^pG^5^ step (Supplementary Figure S8A–E). Collectively, based on model configuration 1, we propose that the Oct4 POU_HD_ homodimerization is likely driven by direct interactions between the N-terminal tails and the CpGpal is recognized by a combination of direct and indirect readout mechanisms.

**Figure 7.**
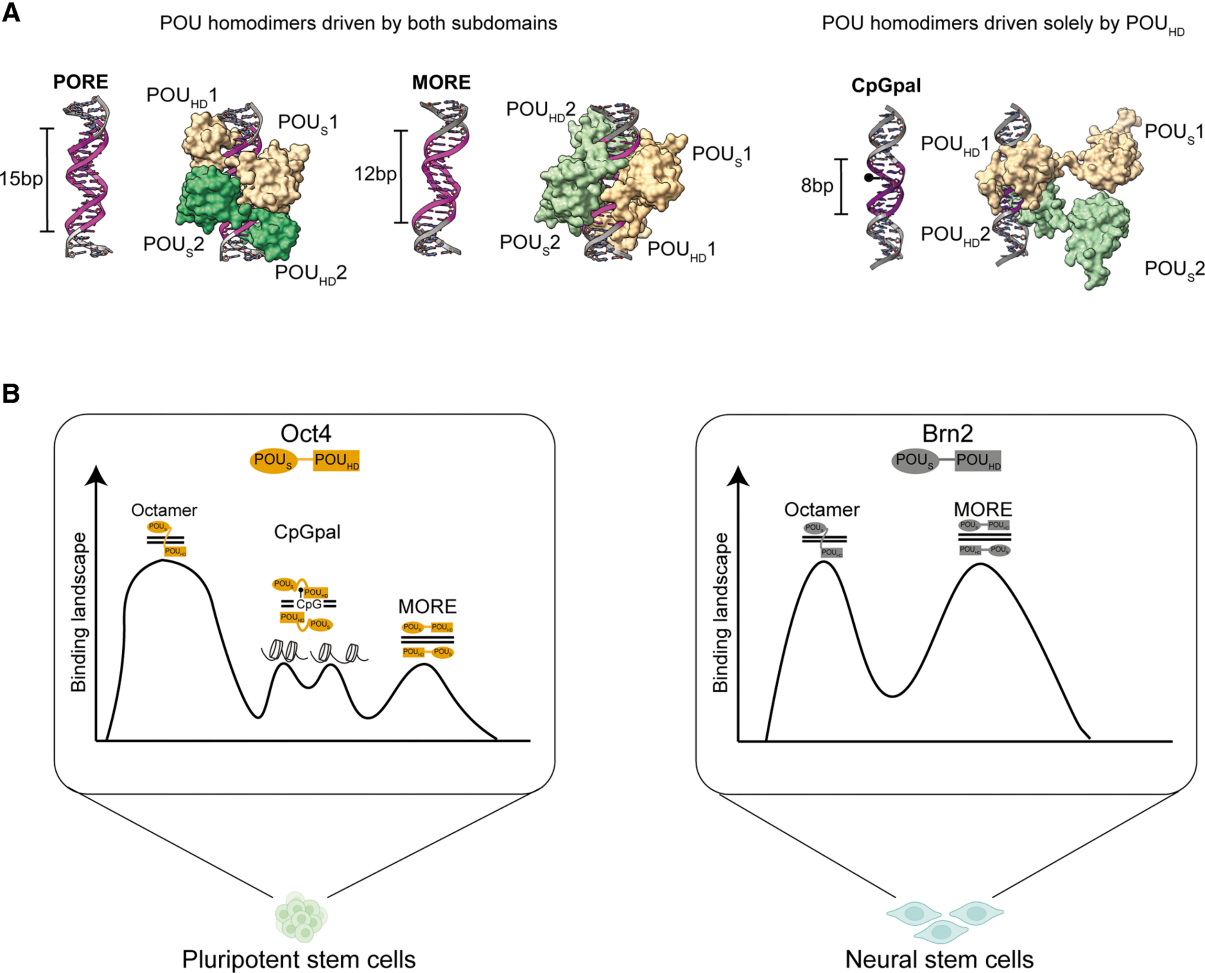
Alternative POU dimers and binding landscapes of Oct4 in pluripotent and Brn2 in neural stem cells. (**A**) Schematic summary of different POU dimer configurations. Contrary to previously studied MORE and PORE elements, Oct4 dimerizes effectively using only one of its two subdomains for the cooperative dimerization. MORE and PORE models are from (20,29). (**B**) Oct4 binds the methylated CpGpal element specifically in the context of pluripotent cells. Oct4 bound CpGpal sites often remain methylated, and a subset remains closed. The POU_S_ and POU_HD_ subdomains are shown as an oblong and rectangle. Oct4 is colored in yellow and Brn2 is grey. Peak height schematizes binding preference.

It is desirable to obtain experimental structures of Oct4/CpGpal complexes in the future as structural modeling has obvious limitation. Yet, our structural models guided by available crystal structures and verified by MD simulations provide a highly plausible conformation as to how Oct4 binds the CpGpal. These models help us to understand why the isolated POU_HD_ dimerizes more effectively on the methylated CpGpal than the full POU domain. The detached POU_S_ can only co-exist in a limited number of conformations, with a majority subject to steric crowding which decreases conformational entropy (100). Removal of the POU_S_ eliminates this negative component to the binding energy leading to increased cooperativity (Figure 5C). Together, structural analysis rationalizes why the homeodomain alone is the main driver for dimerization on the CpGpal. This unique configuration is unlike the POU_S_-POU_HD_ contacts mediating dimer formation on MORE and PORE (Figure 7A). Rather, the POU_S_ is detached from the DNA in this binding mode and could engage in co-factor recruitment, condensate formation, or the sampling of new binding sites.

## DISCUSSION

Most transcription factors have a well-defined consensus binding motif to which they bind with high affinity and specificity. For a subset of factors, alternative or ‘secondary’ binding motifs have been reported which are often related to the dominant primary motif and differ at only 2–3 variant positions (101,102). Transcription factors with multiple DNA binding domains such as Oct4 are predisposed to recognize alternative binding sites that differ more considerably. Alternative binding modes not only expand the repertoire of genomic targets but also enable context-dependent regulatory outcomes. Indeed, for Oct4 and POU family factors, post-translational modifications (103) and interactions with partner factors (16,95,104,105) regulate the choice of DNA binding sites. Hence, alternative DNA binding sites can be thought of as allosteric ligands that change the behavior and function of the associated transcription factor. Here, we defined and characterized a binding configuration of Oct4 that exclusively depends on the POU_HD_. The homodimerization of POU factors on palindromic binding sites has been described for most members of this family in the context of different cell types. For neural POU3 factors, the homodimerization on the MORE appears to be the predominant mode of DNA recognition (33). Yet, in this configuration, both POU_S_ and POU_HD_ are bound to DNA and engage in direct protein-protein interactions (Figure 6B, Supplementary Figure S7D) (43,93). However, in the context of the CpGpal, the POU_S_ is not required for binding and is likely detached from the DNA (Figure 6 D-F, Supplementary Figure S8A–C). This is surprising as previous work suggested that in the context of Octamer elements within nucleosome core particles, the POU_S_ is more critical for binding as the POU_HD_ was invisible in the cryo-EM structure (21). However, the binding of the POU_S_ was stabilized through interactions with Sox2. In the context of the CpGpal, we show that the POU_HD_ of Oct4 is necessary and sufficient for binding.

What could be the significance of a binding mode where one of two DNA binding domains is available for other molecular interactions? We envisage three possible scenarios. First, multi-domain TFs can concurrently bind remote segments of genomic DNA (Supplementary Movie S1-2). This activity could facilitate the scanning of the genome using an intersegmental transfer mechanism (106). Simultaneous binding of distant DNA sites could also lead to the clustering of cis-regulatory elements within regions of high transcriptional activity. Second, transcription factors cluster into dense nuclear speckles and form transcription factories with high activity also called super-enhancers (107). It has been proposed that condensate formation and liquid-liquid phase separation (LLPS) is a key mechanism for the formation of these nuclear aggregates (35). The intrinsically disordered transactivation domains are believed to be the key drivers for the assembly process. Yet, recent studies reported that the structured DNA binding domain can engage in condensate formation (108,109). Oct4 has been shown to drive LLPS *in vitro* which is strongly affected by the spacing of DNA binding sites (110). Therefore, the detached POU_S_ in the context of the CpGpal may influence this behavior and contribute to the assembly of nuclear condensates. Third, the detached POU_S_ may serve as a globular protein-protein interaction module. The structural basis for the interaction of the POU_S_ with Sox2, and Sox17 as well as during homotypic dimerization is known and mediated through specific and conserved interfaces (94,95). Therefore, the detached POU_S_ might engage in specific molecular partnerships and recruit epigenetic effectors or chromatin remodelers.

One interesting possibility is that Oct4 may be directly involved in the maintenance or *de novo* methylation of CpGpal sites. A proteomics analysis reported DNMT3A as an interaction partner of Oct4 in ESCs (111). Future studies should verify the nature of this interaction and its relevance for CpG methylation in pluripotent cells and the germline. CpG methylation is an evolutionarily ancient epigenetic modification. Yet, several eukaryote species such as yeast, insect, and nematode have virtually no 5mC (3). In mammals, dense CpG islands (CGI) are often in promoters that are hypomethylated while isolated CpGs are often hypermethylated (112). The vulnerability of 5mCs for deamination leading to C > T transitions led to an evolutionary underrepresentation of CpGs in the mammalian genome outside of CGIs (113,114). Mice and humans undergo two major waves of erasure and re-establishment of CpG methylation (Supplementary Figure S3) (3). One wave happens in the zygote immediately succeeding fertilization and another wave occurs after the specification of the germline (2,115). Oct4 becomes abundantly expressed at the 8-cell stage, in pluripotent cells and is thereafter restricted to the germ line (81,82), where it is critical in the development of primordial germ cells (PGCs) (12). The CpGpal represents an intriguing and very unconventional CpG element. A hallmark property is that it remains largely methylated whilst most other non-CGI CpG sites become hypomethylated during reprogramming towards naïve pluripotent cells (Figure 2D). This suggests that the methylation of the CpGpal is actively maintained raising questions as to its function in early development. What could then be the functional relevance of the evolutionarily unstable CpGpal and its association with Oct4? The demethylation waves in the early embryo seen in mammals are not conserved in all vertebrates. For example, the germ cells of zebrafish do not undergo global demethylation of CpGs (116). Coincidentally, the POU5 gene class emerged rather recently in jawed vertebrates presumably from an ancestral POU3 gene (about 400 million years ago) (23–25). In zebrafish, Oct4 is a key driver of the zygotic genome activation within the context of a largely methylated genome (117,118). Here, we find that only Oct4 effectively dimerizes on the CpGpal but the POU3 factor Brn2 does not (Figure 4B-E). Consistently, only Oct4 but no other POU factor associates with the CpGpal in cells in a significant manner (Figure 4F-G). Hence, binding to the CpGpal is a unique feature of the evolutionarily young Oct4 but not of other more deeply conserved POU factors. In sum, Oct4 can distinctively form cooperative homodimers through its POU_HD_ domain to methylated CpGpal sequences in pluripotent cells. Unlike, its POU3 family members like Brn2 that cannot engage the motif in cells (Figure 7A, B). The emergence of POU5 factors might have occurred within non-vertebrate organisms where demethylation waves are uncommon. Therefore, the ability to bind methylated DNA elements might be a key requirement for germline specifiers in these species. Future work should compare the role of Oct4 during the germline development of species with and without demethylation. This will clarify whether Oct4 is actively involved in the maintenance of methylation within the germline of certain species.

Oct4 was designated as a pioneer transcription factor for its ability to bind nucleosomes *in vitro* and closed chromatin during pluripotency reprogramming (11,119). Whether Oct4 directly drives chromatin opening or rather assists other pioneer factors such as Sox2 to activate the pluripotency network remains debatable (91,30,120). When bound to the CpGpal, Oct4 appears to neither induce substantial demethylation nor chromatin opening. This suggests that Oct4 could exert regulatory roles in the context of compact and methylated chromatin conventionally deemed repressive. Hence, the pioneer transcription factor model may not apply to all Oct4 binding events and depends on the sequence of the DNA binding site.

## DATA AVAILABILITY

Datasets used in this study are listed in Supplementary Table S3. Atomic coordinates and structure factors for the reported crystal structures have been deposited with the Protein Data bank under PDB ID: 7XRC (https://doi.org/10.2210/pdb7XRC/pdb). Raw images (ID: XRD-00080) deposited in XRDa (https://xrda.pdbj.org/). CpGpal coordinates specified in Supplementary Figure S2C are in Supplementary Table S8. Unprocessed gel images, NGS sequences, PDB files of structural models, MD files and other relevant data tables were uploaded onto Zenodo (https://zenodo.org/record/7436073).

## Supplementary Material

gkac1262_Supplemental_Files
