# Supplementary material for: The homeodomain of Oct4 is a dimeric binder of methylated CpG elements
Source: Nucleic Acids Res. 2023 Jan 12;51(3):1120–38. doi: 10.1093/nar/gkac1262 (PMC9943670; doi:10.1093/nar/gkac1262)
Supplement: gkac1262_Supplemental_Files [file gkac1262_supplemental_files.zip › Supplementary_Figures_Tables_revision2_v3.pdf]

Supplementary information for
The homeodomain of Oct4 is a dimeric binder of methylated CpG elements

Daisylyn Senna Tan[1], Shun Lai Cheung[1], Ya Gao[1], Maike Weinbuch[1,2], Haoqing Hu[1], Liyang Shi[3], Shih-Chieh Ti[1], Andrew P Hutchins[3] ,Vlad Cojocaru[4,5], and Ralf Jauch[1,6]*

Affiliations:

1. School of Biomedical Sciences, Li Ka Shing Faculty of Medicine, The University of Hong Kong, Hong Kong SAR, China
2. Institute for Molecular Medicine, Ulm University, Germany
3. Shenzhen Key Laboratory of Gene Regulation and Systems Biology, Department of Biology, School of Life Sciences, Southern University of Science and Technology, Shenzhen, 518055, China
4. STAR-UBB Institute, Babeș-Bolyai University, Cluj-Napoca, Romania
5. Computational Structural Biology Group, Utrecht University, the Netherlands
6. Centre for Translational Stem Cell Biology, Hong Kong SAR, China

*Correspondence to: Ralf Jauch

Tel: +852 3917 9511; Fax: +852 28559730

Email: ralf@hku.hk

SUPPLEMENTARY FIGURES

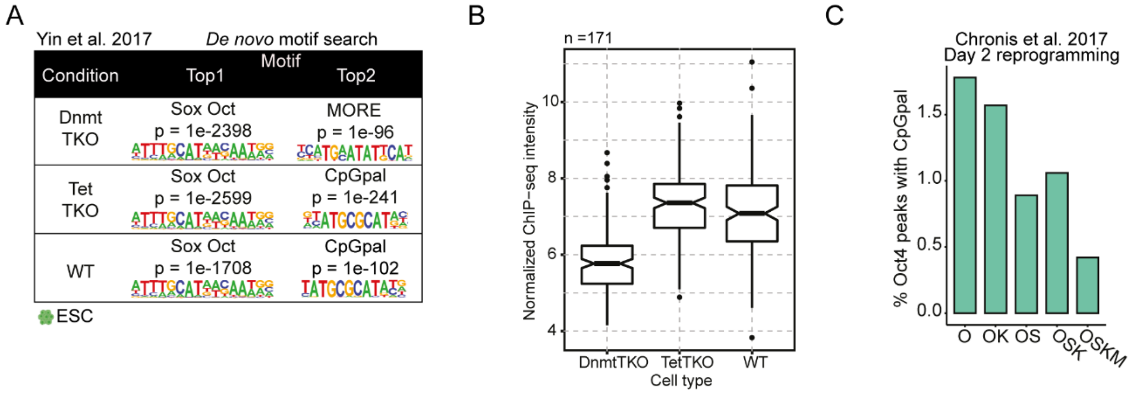

Figure S1 Enrichment of CpGpal sites in pluripotent stem cells

A. Top 2 enriched *de novo* motifs in Oct4 ChIP-seq data from wild type (WT) as well as in hypomethylated (Dnmt-TKO) or hypermethylated (Tet-TKO) mouse ESCs
B. Box plots showing ChIP-seq peak intensities normalized by MAnorm2 at Oct4/CpGpal sites in indicated ESC lines
C. Percentage of Oct4 bound sites containing the CpGpal expressing indicated combinations of OSKM factors at day 2 of iPSC reprogramming

(B) Boxes represent the interquartile with a median line. Whiskers indicate last values within 1.5 times the interquartile range. Notches display 95% confidence interval around the median

Abbreviations

Dnmt-TKO: DNA methyltransferases Dnmt1, Dnmt3a, and Dnmt3b – triple knockout

Tet-TKO: Tet1, Tet2, and Tet3 – triple knockout

ESC: Embryonic Stem Cells, O: Oct4, S: Sox2, K: Klf4, M: c-Myc

iPSC: induced Pluripotent Stem Cells

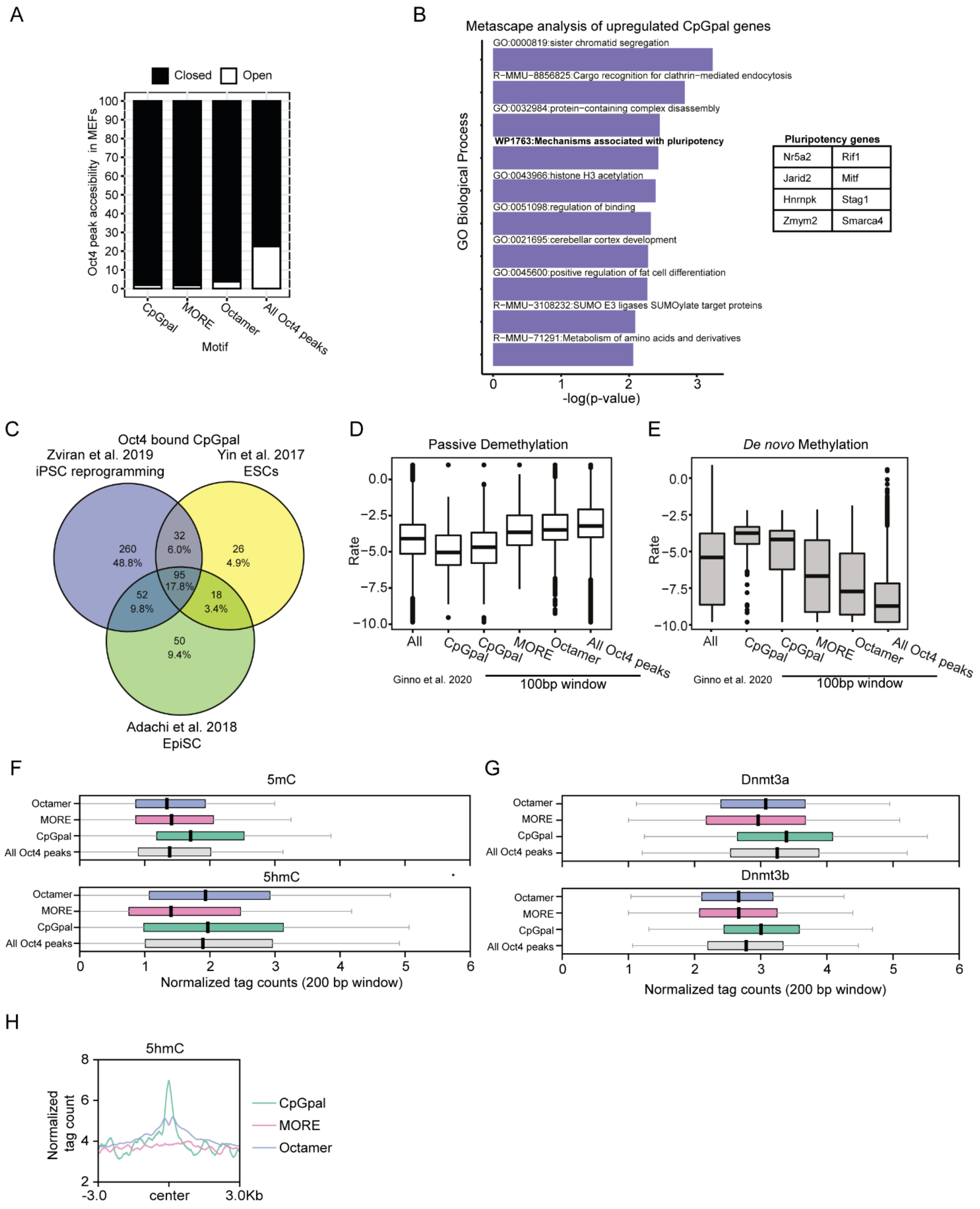

A
Closed
Open
Oct4 peak accesibility in MEFs
Motif
CpGpal
MORE
Octamer
All Oct4 peaks
B
Metascape analysis of upregulated CpGpal genes
GO:0000819:sister chromatid segregation
R-MMU-8856825:Cargo recognition for clathrin-mediated endocytosis
GO:0032984:protein-containing complex disassembly
WP1763:Mechanisms associated with pluripotency
GO:0043966:histone H3 acetylation
GO:0051098:regulation of binding
GO:0021695:cerebellar cortex development
GO:0045600:positive regulation of fat cell differentiation
R-MMU-3108232:SUMO E3 ligases SUMOylate target proteins
R-MMU-71291:Metabolism of amino acids and derivatives
GO Biological Process
-log(p-value)
Pluripotency genes
Nr5a2
Rif1
Jarid2
Mitf
Hnrnpk
Stag1
Zmym2
Smarca4
C
Oct4 bound CpGpal
Zviran et al. 2019
iPSC reprogramming
Yin et al. 2017
ESCs
Adachi et al. 2018
EpiSC
260 48.8%
32 6.0%
26 4.9%
95 17.8%
52 9.8%
18 3.4%
50 9.4%
D
Passive Demethylation
Rate
All
CpGpal
CpGpal
MORE
Octamer
All Oct4 peaks
Ginno et al. 2020
100bp window
E
De novo Methylation
Rate
All
CpGpal
CpGpal
MORE
Octamer
All Oct4 peaks
Ginno et al. 2020
100bp window
F
5mC
5hmC
Octamer
MORE
CpGpal
All Oct4 peaks
Normalized tag counts (200 bp window)
G
Dnmt3a
Dnmt3b
Octamer
MORE
CpGpal
All Oct4 peaks
Normalized tag counts (200 bp window)
H
5hmC
Normalized tag count
-3.0
center
3.0Kb
CpGpal
MORE
Octamer

Figure S2 Characterisation of CpGpal sites

A. The accessibility in MEFs for indicated categories of Oct4 binding sites
B. Top gene ontology (GO) terms for upregulated genes linked to Oct4/CpGpal (Figure 2I). Eight pluripotency genes are listed
C. Venn diagram showing the intersection of Oct4/CpGpal motif coordinates between three indicated studies

D-E Rate of (D) passive demethylation and (E) *de novo* methylation at Oct4 binding sites with indicated motifs determined in mouse ESCs (1)

F. Boxplots of 5mC or 5hmC signals at indicated Oct4 bound motifs
G. Boxplots of Dnmt3a or Dnmt3b ChIP-seq signals at indicated Oct4 bound motifs
H. Line plot of 5hmC signals at a 6kbp window centered at indicated Oct4 peak categories

(D-G) Boxes represent the interquartile with a median line. Whiskers indicate last values within 1.5 times the interquartile range.

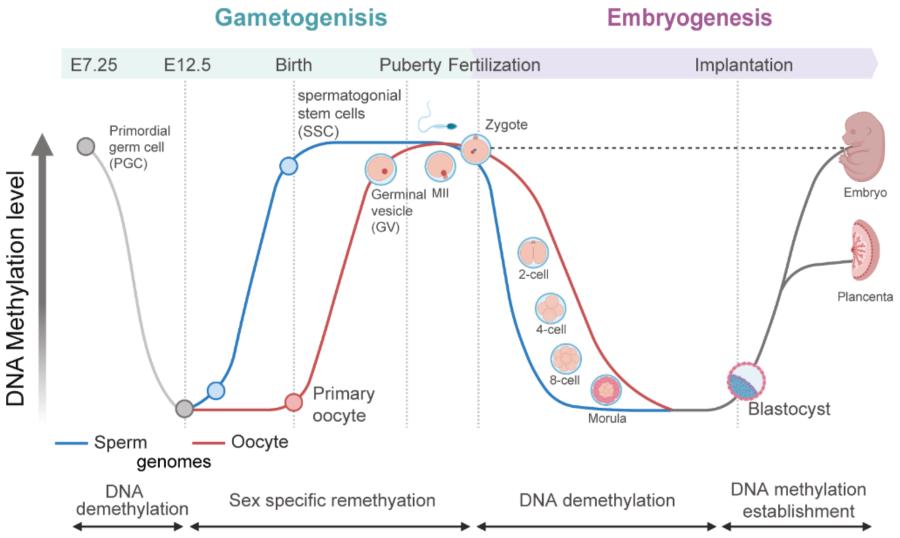

Figure S3. DNA methylation dynamics during gametogenesis and embryogenesis

Schematic diagram showing the methylation dynamic during development (Biorender) which is referenced from (2). Cell types analyzed for the methylation of CpGpal site in cells (Figure 3F) are marked (Zygote, 2-cell, 4-cell, 8-cell, morula, PGC, SSC, GV).

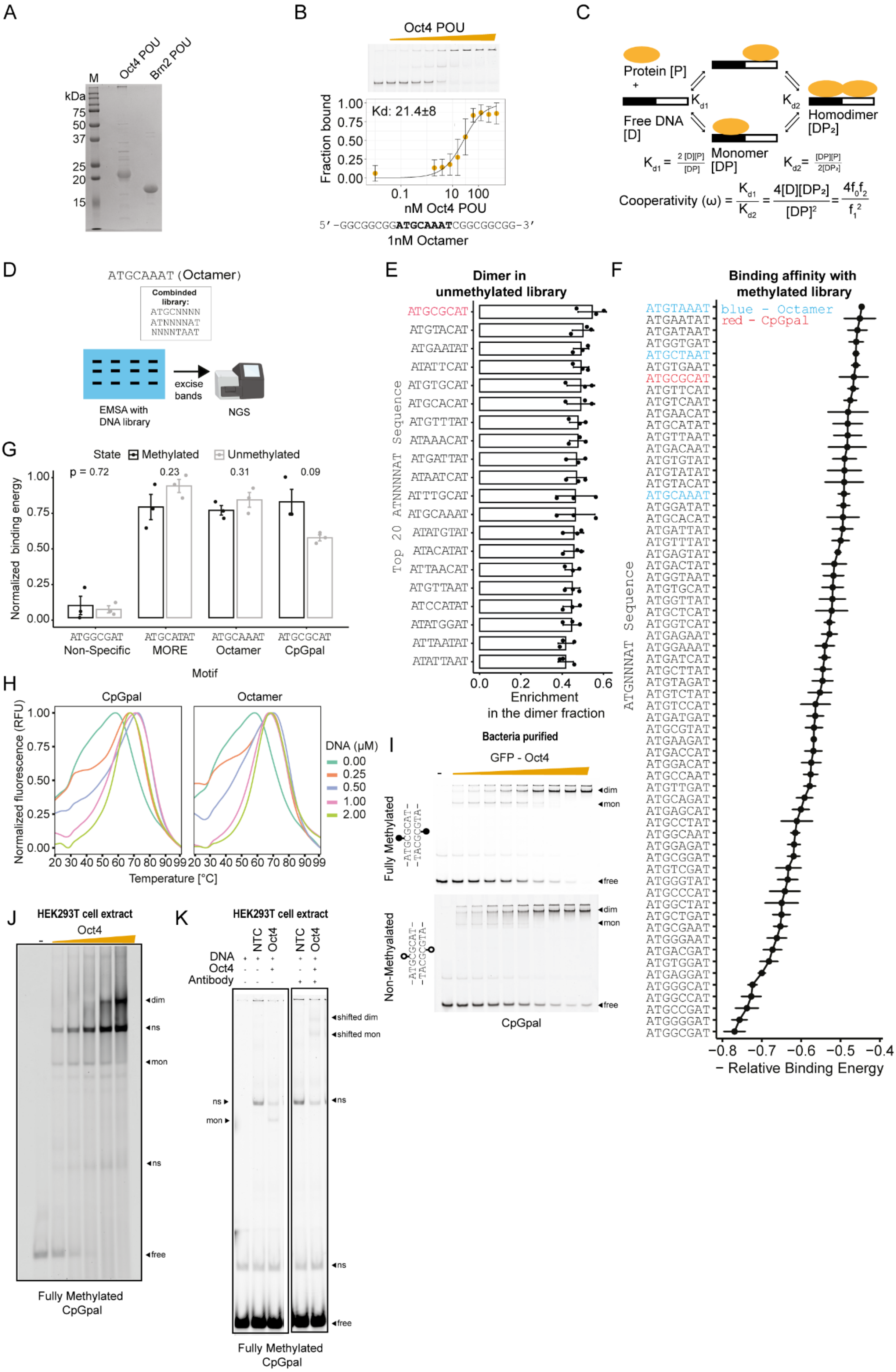
A
M
Oct4 POU
Brn2 POU
kDa
75
50
37
25
20
15
B
Oct4 POU
Kd: 21.4±8
Fraction bound
1.00
0.75
0.50
0.25
0.00
0.1
10
100
nM Oct4 POU
5'-GGCGGCGGATGCAAATCGGCGGCGG-3'
1nM Octamer
C
Protein [P]
+
Free DNA
[D]
K_{d1}
K_{d2}
Homodimer
[DP₂]
Monomer
[DP]
$K_{d1} = \frac{2[D][P]}{[DP]}$
$K_{d2} = \frac{[DP][P]}{2[DP_2]}$
Cooperativity (ω) = $\frac{K_{d1}}{K_{d2}} = \frac{4[D][DP_2]}{[DP]^2} = \frac{4f_0f_2}{f_1^2}$
D
ATGCAAAT (Octamer)
Combinded library:
ATGCNNNN
ATNNNNAT
NNNNTAAT
EMSA with DNA library
excise bands
NGS
E
Dimer in unmethylated library
Top 20 ATNNNNAT Sequence
ATGCGCAT
ATGTACAT
ATGAATAT
ATATTCAT
ATGTGCAT
ATGCACAT
ATGTTTAT
ATAAACAT
ATGATTAT
ATAATCAT
ATTTGCAT
ATGCAAAT
ATATGTAT
ATACATAT
ATTAACAT
ATGTTAAT
ATCCATAT
ATATGGAT
ATTAATAT
ATATTAAT
0.0
0.2
0.4
0.6
Enrichment in the dimer fraction
F
Binding affinity with methylated library
blue - Octamer
red - CpGpal
ATGNNNAT Sequence
ATGTAAAT
ATGAATAT
ATGATAAT
ATGGTGAT
ATGCTAAT
ATGTGAAT
ATGCGCAT
ATGTTCAT
ATGTCAAT
ATGAACAT
ATGCATAT
ATGTTAAT
ATGACAAT
ATGTGTAT
ATGTATAT
ATGTACAT
ATGCAAAT
ATGGATAT
ATGCACAT
ATGATTAT
ATGTTTAT
ATGAGTAT
ATGACTAT
ATGGTAAT
ATGTGCAT
ATGGTTAT
ATGCTCAT
ATGGTCAT
ATGAGAAT
ATGGAAAT
ATGATCAT
ATGCTTAT
ATGTAGAT
ATGTCTAT
ATGTCCAT
ATGATGAT
ATGCGTAT
ATGAAGAT
ATGACCAT
ATGGACAT
ATGCCAAT
ATGTTGAT
ATGCAGAT
ATGAGCAT
ATGCCTAT
ATGGCAAT
ATGGAGAT
ATGCGGAT
ATGTCGAT
ATGGGTAT
ATGCCCAT
ATGGCTAT
ATGCTGAT
ATGCGAAT
ATGGGAAT
ATGACGAT
ATGTGGAT
ATGAGGAT
ATGGGCAT
ATGGCCAT
ATGCCGAT
ATGGGGAT
ATGGCGAT
−0.8
−0.7
−0.6
−0.5
−0.4
− Relative Binding Energy
G
State
Methylated
Unmethylated
Normalized binding energy
1.00
0.75
0.50
0.25
0.00
p = 0.72
0.23
0.31
0.09
ATGGCGAT
Non-Specific
ATGCATAT
MORE
ATGCAAAT
Octamer
ATGCGCAT
CpGpal
Motif
H
CpGpal
Octamer
Normalized fluorescence (RFU)
1.00
0.75
0.50
0.25
0.00
20 30 40 50 60 70 80 90 99
Temperature [°C]
DNA (μM)
0.00
0.25
0.50
1.00
2.00
I
Bacteria purified
GFP - Oct4
-
Fully Methylated
-ATGCGCAT-
-TACGCGTA-
dim
mon
free
Non-Methylated
CpGpal
J
HEK293T cell extract
Oct4
-
dim
ns
mon
ns
free
Fully Methylated
CpGpal
K
HEK293T cell extract
NTC
Oct4
NTC
Oct4
DNA
Oct4
Antibody
shifted dim
shifted mon
ns
mon
ns
ns
free
Fully Methylated
CpGpal

Figure S4. DNA binding preferences of Oct4

A. SDS-PAGE gel of purified Oct4 and Brn2 POU domains
B. Binding of Oct4 POU to 1nM Octamer DNA. Representative EMSA titrations from 3 replicate experiments (top) and binding isotherm (bottom) for fractional binding at increasing concentration of Oct4 POU(1-500nM). The equilibrium dissociation constant (Kd) is shown as mean +/- sd
C. Schematic diagram highlighting the approach to calculate the cooperativity of homodimer formation on DNA elements as originally detailed in (3). Monomeric binding ensues to either one of two identical palindromic half sites followed by homodimer complex formation by binding to the remaining site. Kd_1 and Kd_2 are the equilibrium dissociation constants for monomer or dimer formation. [D], [P], [DP] and [DP_2] are the fractional concentrations of the free DNA, protein, monomer–DNA complex and dimer-DNA complex, respectively. These concentrations can be estimated by separating the microstates in a native gel where the fractions of free DNA (f_0), monomerically bound DNA (f_1), and dimerically bound DNA (f_2) can be quantified. The cooperativity factor (ω) can then be calculated directly from these factional contributions.
D. Schematic of the Spec-seq experiment. Three indicated degenerate libraries were mixed in the experiment with unmethylated DNA
E. Bar plots of the top 20 ATNNNNAT sequences ranked by fractional enrichment within the dimer band. Data shown as mean ±sd. Fractional enrichment = $\#S_{dim}$ / ($\#S_{dim}$+$\#S_{mon}$ +$\#S_{free}$), where $\#S_{dim}$, $\#S_{mon}$, and $\#S_{free}$ are the numbers of reads of each sequence (S) in the dimer, monomer and free DNA bands, respectively.
F. Relative binding energy for all 64 variants in methylated ATGNNNAT libraries ranked from low to high binding energies. The relative binding energies are represented in units of kT, where k is the Boltzmann constant and T is temperature. Energies are calculated by $\ln(\#S_{unbound}/\#S_{bound})$, where $\#S_{unbound}$ and $\#S_{bound}$ are the numbers of reads each sequence (S) according to (4)
G. Bar plots comparing the Minmax normalized binding energies of relevant motif sequences from binding experiments in E and F
H. Normalized thermal unfolding curves of Oct4 POU bound to CpGpal or Octamer DNA
I. Representative EMSA titrations from 2 replicate experiments with 50nM Cy5 labeled CpGpal DNA probes and increasing concentrations of the GFP labelled Oct4 full-length protein purified from bacteria (25-800nM)
J. Representative EMSA titrations from 2 replicate experiments using increasing concentration of full-length Oct4 protein from HEK293T whole cell extracts and fully methylated CpGpal
K. EMSA using full-length Oct4 protein from HEK293T whole cell extracts and fully methylated CpGpal with (right) and without (left) Oct4 antibodies. Bands with Oct4/CpGpal that are super shifted and labeled as shifted

(I-K) Arrows are highlighting free DNA (free) or shifted DNA bands bound to monomeric (mon) or dimeric (dim) protein.

(F-G) Data are shown as mean ±SEM of three EMSA replicates with increasing concentrations of Oct4 POU. p-values were determined from unpaired Student’s t test.

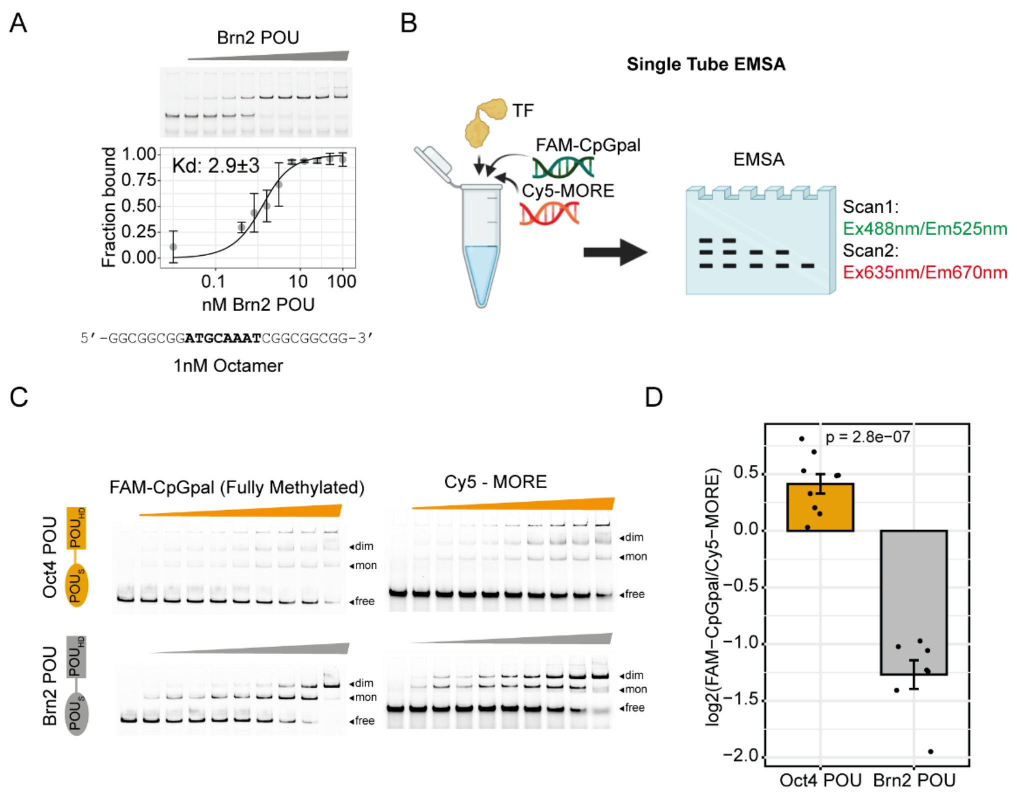

Figure S5. The DNA binding preferences of Brn2 differ from Oct4

A. Binding of Brn2 POU to 1nM Octamer DNA. Representative EMSA titration from 3 replicate experiments (top) and binding isotherm (bottom) for fractional binding at increasing concentration of Brn2 POU (0.4-100nM). The calculated equilibrium dissociation constant (Kd) is shown as mean +/- sd
B. Schematic of the single tube EMSA experiment. Differently labelled DNA probes (FAM-CpGpal or Cy5-MORE) were simultaneously incubated with the purified proteins of Oct4 POU or Brn2 POU in a single tube reaction. Complexes were separated on native gels and successively imaged using FAM [Ex488nm/Em525nm] and Cy5[Ex635nm/Em670nm] channels.
C. Representative gels from 2 replicate experiments of single tube EMSAs described in B, containing either Oct4 POU or Brn2 POU in combination with FAM-CpGpal (fully methylated) and Cy5-MORE.
D. Bar plots representing differences in DNA-binding preference as determined by the single tube EMSA experiments in C. The dimeric fractions of Brn2 and Oct4 when bound to FAM-CpGpal (fully methylated) or Cy5-MORE DNA elements are shown as log2 ratios. Data are shown as mean ±SEM from with p-values determined from unpaired Student's t test (n = 6-9 data points/lanes from two EMSA replicates) Only lanes where the fractional contribution of each of the three bands was at least 0.05 were included.

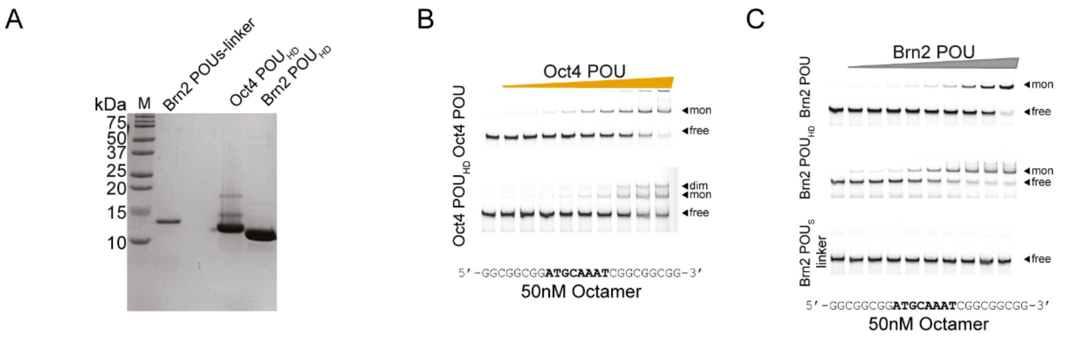

D

Proteins	pI	Charge at pH 7.5
Oct4 POU	9.5	10.9
Brn2 POU	9.0	5.9
Oct4 POU_{HD}	11.0	9.9
Brn2 POU_{HD}	10.1	7.9
Oct4 POU_{S} linker	7.6	0.1
Brn2 POU_{S} linker	5.8	-1.8

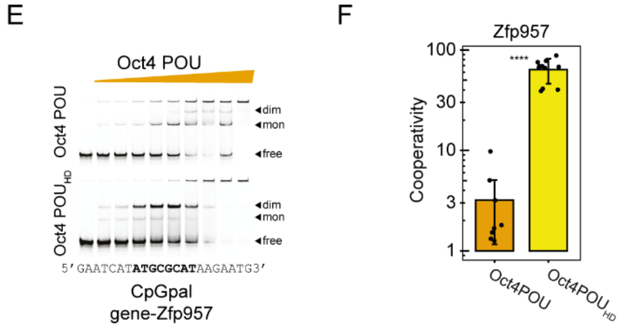

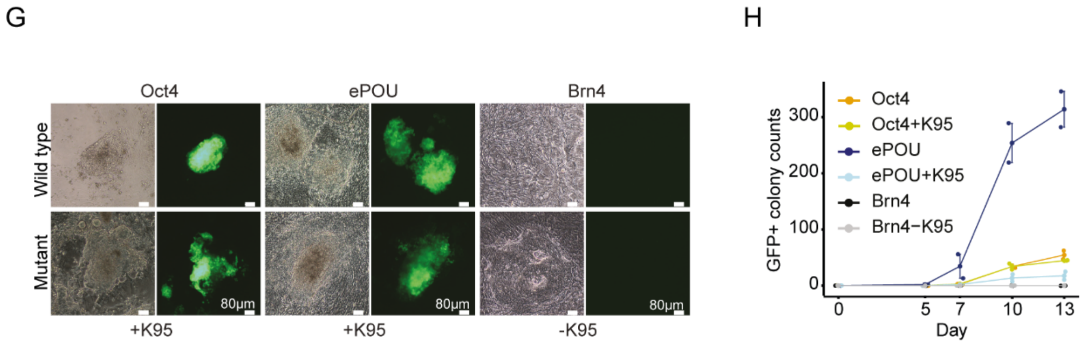

Figure S6. The isolated Oct4 POU_{HD} alone binds the CpGpal regardless of methylation

A. SDS-PAGE gel of purified subdomains of Oct4 POU (POU_{HD})and Brn2 POU (POU_{s} and POU_{HD})

B-C Representative EMSA titrations from 2 replicate experiments of (B) Oct4 POU DBD and POU_{HD} as well as (C) Brn2 POU DBD, POU_{S} linker and POU_{HD} on 50nM Cy5 labelled Octamer DNA probes

D. Protein properties of constructs in A. Isoelectric point (pI) and net charge of protein at pH 7.5 were calculated using Protpi (https://www.protpi.ch/Calculator/ProteinTool)

E. Representative EMSA titrations from 2 replicate experiments of of the Oct4 POU domain and the POU_{HD} on a genomic sequence with the CpGpal motif near *Zfp957* gene

F. Calculation of cooperativity factors (ω) for EMSAs in E (n =7-10)

G. Representative Oct4-GFP positive colonies at day 11 of mouse iPSC reprogramming of conditions specified in Figure 5D

H. Counts of GFP+ iPSC colonies from two independent experiments at different days of reprogramming. (n = 2) Date are shown as mean ± range

(B, C, E & F) Arrows are highlighting free DNA (free), monomer (mon) or dimer (dim) bound DNA bands. Data are shown as mean ±sd. p-value were determined from unpaired Student’s t test.

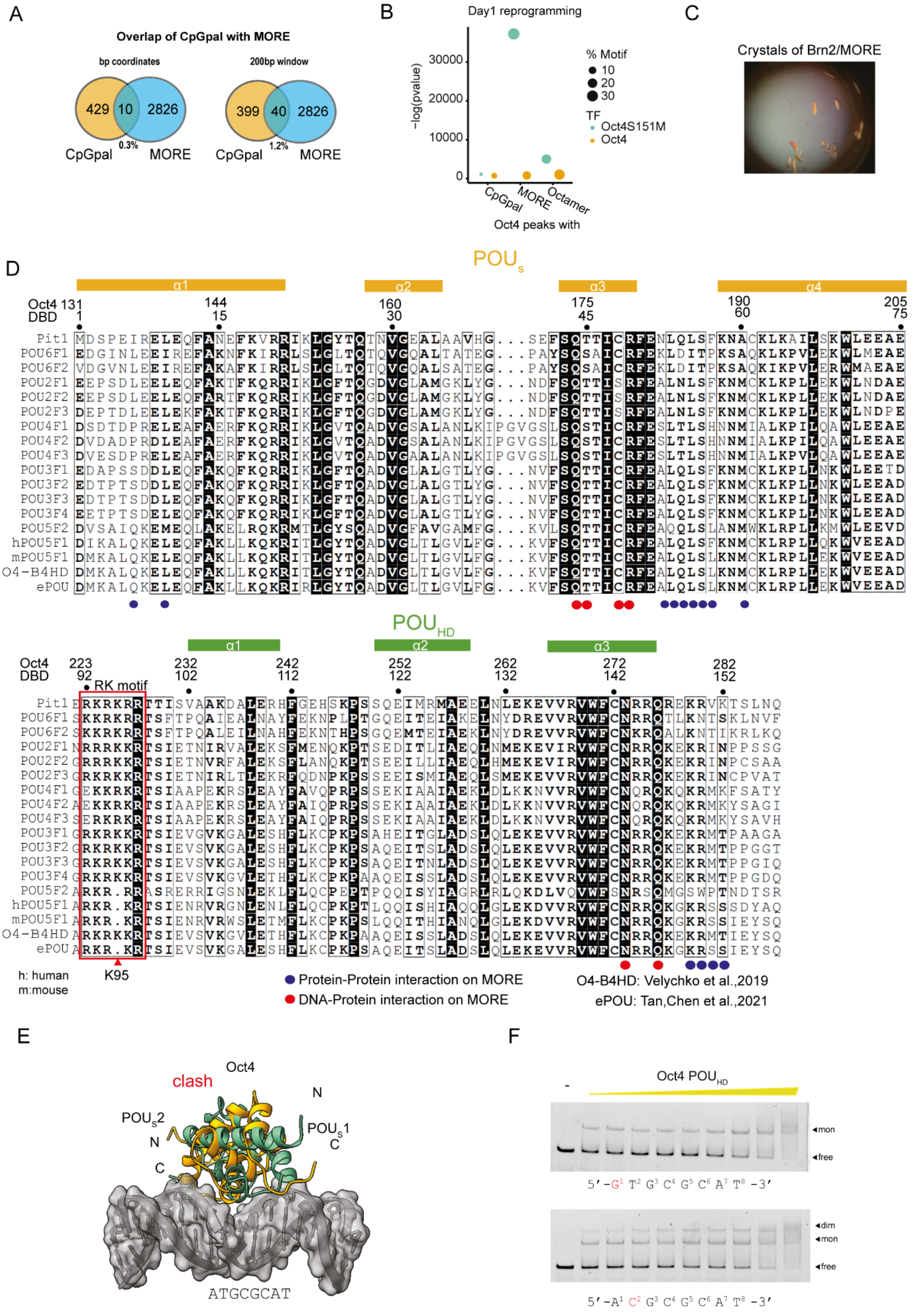

A
Overlap of CpGpal with MORE
bp coordinates
429
10
2826
0.3%
CpGpal
MORE
200bp window
399
40
2826
1.2%
CpGpal
MORE
B
Day1 reprogramming
-log(pvalue)
30000
20000
10000
0
% Motif
10
20
30
TF
Oct4S151M
Oct4
CpGpal
MORE
Octamer
Oct4 peaks with
C
Crystals of Brn2/MORE
D
POUs
α1
α2
α3
α4
Oct4 131 DBD 1
144 15
160 30
175 45
190 60
205 75
Pit1
POU6F1
POU6F2
POU2F1
POU2F2
POU2F3
POU4F1
POU4F2
POU4F3
POU3F1
POU3F2
POU3F3
POU3F4
POU5F2
hPOU5F1
mPOU5F1
O4-B4HD
ePOU
POUHD
Oct4 223 DBD 92
232 102
242 112
252 122
262 132
272 142
282 152
RK motif
K95
h: human
m:mouse
Protein-Protein interaction on MORE
DNA-Protein interaction on MORE
O4-B4HD: Velychko et al.,2019
ePOU: Tan,Chen et al.,2021
E
Oct4
clash
N
POUs2
POUs1
N
C
C
ATGCGCAT
F
-
Oct4 POUHD
mon
free
5′-G1 T2 G3 C4 G5 C6 A7 T8 -3′
dim
mon
free
5′-A1 C2 G3 C4 G5 C6 A7 T8 -3′

Figure S7. The 1.9 Å crystal structure of Brn2 POU dimers bound to MORE DNA

A. Venn diagrams showing the intersection between Oct4 bound CpGpal and MORE motif coordinates (bottom left) and their co-occurrence within 200 bp windows (bottom right)
B. Enrichment of CpGpal, Octamer, and MORE motifs in Oct4 or Oct4SM ChIP-seq peaks. Oct4SM is a Oct4 Ser151Met mutant with enhanced dimerization on the MORE (5).
C. Photographs of crystals of the Brn2/MORE ternary complex used for diffraction analysis
D. Alignment of all mouse POU family members, human Oct4 (hPOU5F1) and the Oct4 mutants tested in reprogramming. α-helices are marked with bars, blue circles indicate MORE protein-protein interaction sites and red circles mark residues engaged in base-specific binding to MORE DNA. The numbering scheme refers to the mouse full-length protein and the POU DBD numbering scheme used in (5,6)
E. Structural models of Oct4 POU_S bound to CpGpal generated from MORE models after removal of the central ATAT with the POU_S retained showing incompatible clashes
F. Representative EMSA titrations from 2 replicate experiments of Oct4 POU_{HD} with FAM-labelled DNA probes of CpGpal with the specific AT nucleotides predicted to be recognized in the models mutated. Arrows are highlighting free DNA (free), monomer (mon) or dimer (dim) bound DNA bands.

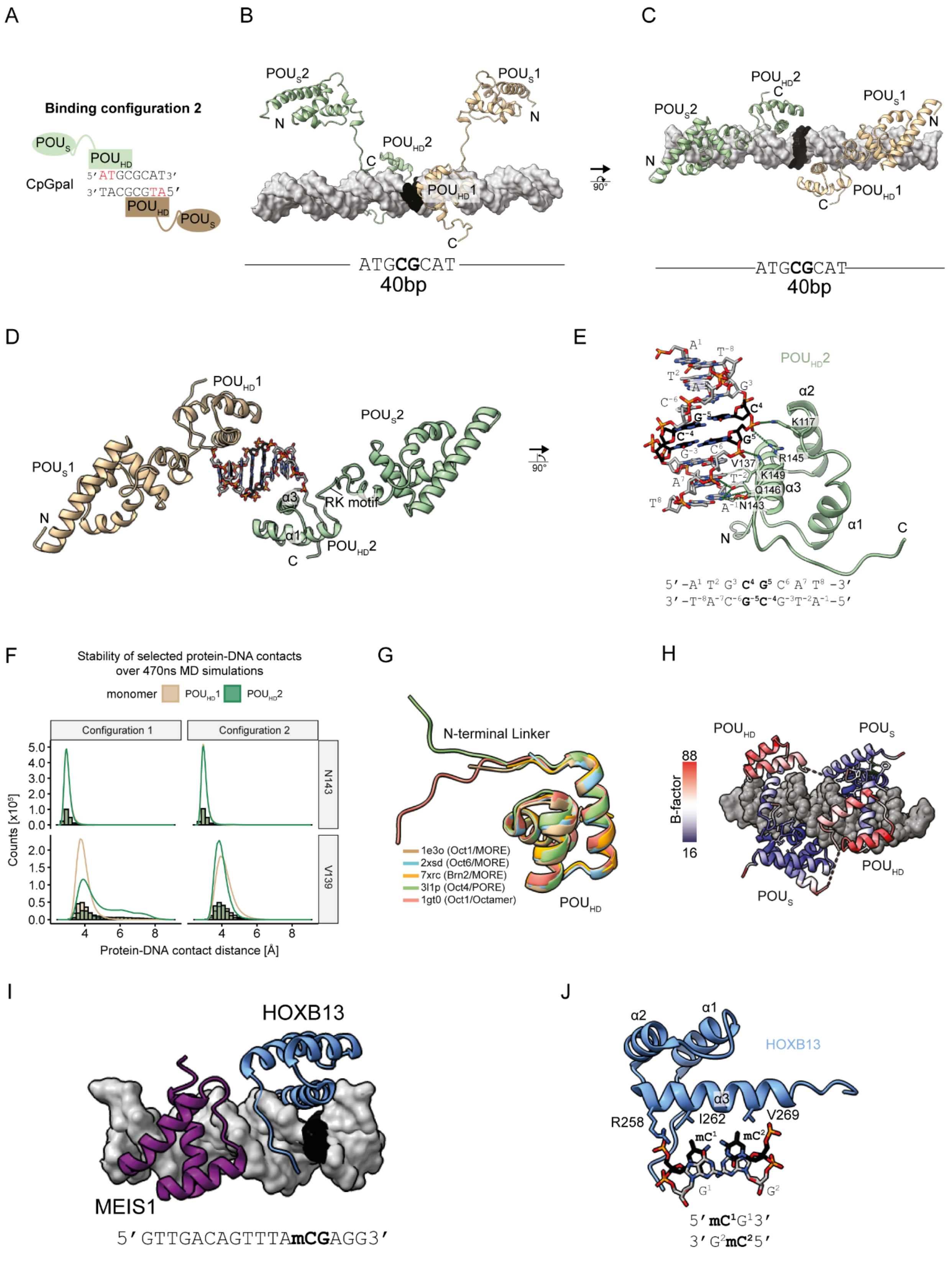
A
B
C
Binding configuration 2
CpGpal
5′ATGCGCAT3′
3′TACGCGTA5′
ATGCGCAT
40bp
D
E
5′-A1 T2 G3 C4 G5 C6 A7 T8 -3′
3′-T-8A-7C-6G-5C-4G-3T-2A-1-5′
F
Stability of selected protein-DNA contacts over 470ns MD simulations
Configuration 1
Configuration 2
Counts [x10⁵]
Protein-DNA contact distance [Å]
G
N-terminal Linker
1e3o (Oct1/MORE)
2xsd (Oct6/MORE)
7xrc (Brn2/MORE)
3l1p (Oct4/PORE)
1gt0 (Oct1/Octamer)
H
B-factor
I
HOXB13
MEIS1
5′GTTGACAGTTTAmCGAGG3′
J
HOXB13
5′mC1G13′
3′G2mC25′

Figure S8. Structural basis for the binding of Oct4 POU_{HD} to CpGpal

A. Schematic of the Oct4 POU bound to CpGpal in an alternative orientation where POU_{HD} recognizes the AT bp on the opposite strand. Base-specifically bound nucleotides are bound by V149/N143/Q146 and highlighted in red.

B-C Structural models of Oct4 POU bound to CpGpal schematised in A with POU_{HD} showing a dimer configuration mediated solely by DNA whilst the POU_S is detached from the DNA.

D. Focused view of the models in B-C with the CpGpal sequences highlighted with helix 3 of both POU_{HD} shown to insert into the major groove of the DNA.

E. Amino acid interaction of the Oct4 POU_{HD} with the central CpG of CpGpal (ATGCpGCAT) in the models described A-C. Contact residues – of the AT (V139, N143, Q146) and the core CpG of CpGpal (K117, R145, K149) are highlighted for 1 monomer of POU_{HD}

F. Histograms representing distances between two heavy atoms of V139 and N143 with DNA bases demonstrate the stability of protein-DNA contacts during 470 ns MD simulations.

G. A superposition of various POU_{HD} crystal structures illustrates the conformational variability of the N-terminal linker when binding to different DNA elements (Octamer, MORE, and PORE)

H. Brn2 POU homodimer bound to the MORE DNA (PDB:7XRC) colored based on b-factor of each amino acid. Red indicating higher B-factor and more flexible regions and blue indicating lower b-factor

I. The Structure of HOXB13 (a methyl-sensitive homeodomain TF) heterodimer with MEIS1 bound to its methylated consensus sequence [PDB ID: 5EGO]

J. Direct readout of methylated CpG seen in HOXB13.

(A-E) Models and cartoons are showing individual subdomains (POU_S and POU_{HD}) of two molecules of Oct4 DBD (colored in sea green and tan). Models are made with a 40 bp genomic DNA sequence (gene - *Nr5a2*) containing the CpGpal motif at the center, colored in grey. Proteins are shown as ribbons and the DNA as surfaces with the CpG core of the motif highlighted in black. Residues highlighted are in POU domain numbering

SUPPLEMENTARY TABLES

Table S1. DNA oligos and primers used

Name	Forward	Reverse	Mod
**EMSA Oligos used - Forward primers are fluorescently labelled with Cy5 or FAM**			
Octamer	GGCGGCGG**ATGCAAAT**CGGCGGCGG	CCGCCGCCG**ATTTGCAT**CCGCCGCC	
MORE	TCCTC**ATGCATATGCAT**GAGGA	TCCTC**ATGCATATGCAT**GAGGA	
CpGpal	GCCGCCTT**ATGCGCAT**AAGCCGCCGC	GCGGCGGCTT**ATGCGCAT**AAGGCGGC	/i5MedC/ or /i5hxdroxydC/
ZFP957 CpGpal	GAATCAT**ATGCGCAT**AAGAATG	CATTCTT**ATGCGCAT**ATGATTC	
CpGpal Mutant 1	GCCGCCTT**GTGCGCAT**AAGCCGCCGC	GCGGCGGCTT**ATGCGCAC**AAGGCGGC	
CpGpal Mutant 2	GCCGCCTT**ATGCGCGT**AAGCCGCCGC	GCGGCGGCTT**ACGCGCAT**AAGGCGGC	
**Cloning primers (blue: Gateway cloning overhangs, black: template-specific)**			
Oct4 POU_{HD}	GGGGACAAGTTTGTACAAAAAAGCAGGCTTCGAAAACCTGTATTTTCAGGGCTGCAAATCGGAGACCATGGTGC	GGGGACCACTTTGTACAAGAAAGCTGGGTTTATCATTCTCGTTGGGAATACTCAATACT	
Brn2 POUs Linker	GGGGACAAGTTTGTACAAAAAAGCAGGCTTCGAAAACCTGTATTTTCAGGGCGACCCGCACTCGGACGA	GGGGACCACTTTGTACAAGAAAGCTGGGTTTATCAGCGCCCTTGCGCTGCGATCTTG	
Brn2 POU_{HD}	GGGGACAAGTTTGTACAAAAAAGCAGGCTTCGAAAACCTGTATTTTCAGGGCAAACGGAAAAAGCGGACCTCCA	GGGGACCACTTTGTACAAGAAAGCTGGGTTTATCACAGAGTCCCTCCGGGAG	
**Primers for point mutation of Oct4, Brn4 and ePOU**			
Oct4+K95	GCAGGCCCGGAAGAGAAAGAAGCGAACTAGCATTG	CAATGCTAGTTCGCTTCTTTCTCTTCCGGGCCTGC	
ePOU+K95	CAGGCCCGGAAGAGAAAGAAGCGAACGTCCATC	GATGGACGTTCGCTTCTTTCTCTTCCGGGCCTG	
Brn4-K95	CAAGGCCGCAAACGCAAGCGAACCTCCATC	GATGGAGGTTCGCTTGCGTTTGCGGCCTTG	

Table S2. Data collection and refinement statistics.

**Wavelength**	0.9184
**Resolution range**	34.35 - 1.89 (1.96 - 1.89)
**Space group**	C 1 2 1
**Unit cell**	94.35 50.63 69.31 90 129.06 90
**Total reflections**	137395 (13983)
**Unique reflections**	19744 (1951)
**Multiplicity**	7.0 (7.2)
**Completeness (%)**	95.89 (95.80)
**Mean I/sigma(I)**	20.08 (0.88)
**Wilson B-factor**	46.24
**R-merge**	0.04967 (2.605)
**R-meas**	0.05373 (2.807)
**R-pim**	0.02021 (1.037)
**CC1/2**	1 (0.476)
**CC***	1 (0.803)
**Reflections used in refinement**	19672 (1941)
**Reflections used for R-free**	982 (96)
**R-work**	0.2001 (0.4514)
**R-free**	0.2271 (0.4988)
**CC(work)**	0.970 (0.621)
**CC(free)**	0.967 (0.370)
**Number of non-hydrogen atoms**	1568
**macromolecules**	1498
**ligands**	0
**solvent**	70
**Protein residues**	134
**RMS(bonds)**	0.008
**RMS(angles)**	1.19
**Ramachandran favored (%)**	98.46
**Ramachandran allowed (%)**	1.54
**Ramachandran outliers (%)**	0.00
**Rotamer outliers (%)**	0.00
**Clashscore**	4.61
**Average B-factor**	67.33
**macromolecules**	70.41
**solvent**	57.29
**Number of TLS groups**	7

Statistics for the highest-resolution shell are shown in parentheses.

Table S3. Re-analyzed datasets

Study	Accession no.	Data type	Species	Reference
Yin et al, 2017	PRJEB9797 (ENA)	ChIP-seq	*Mus musculus*	(7)
Knaupp et al, 2017	GSE101905	ChIP-seq	*Mus musculus*	(8)
Zviran et al, 2019	GSE102518	ChIP-seq, ATAC-seq, WGBS	*Mus musculus*	(9)
Malik et al, 2019	GSE103980	ChIP-seq	*Mus musculus*	(5)
Chronis et al, 2017	GSE90895	ChIP-seq	*Mus musculus*	(10)
ENCODE (ChIP-seq data for POU2F2 and POU5F1 from the Myers lab)	GSM803397, GSM803438	ChIP-seq	*Homo sapiens*	(11,12)
Shen et al, 2017	GSE85062	ChIP-seq	*Mus musculus*	(13)
Lodato et al, 2013	GSE35496	ChIP-seq	*Mus musculus*	(14)
Mistri et al, 2015	GSE69859	ChIP-seq	*Mus musculus*	(15)
Heinz et al, 2010	GSE21512	ChIP-seq	*Mus musculus*	(16)
Adachi et al, 2018	PRJEB18605 (ENA)	ChIP-seq	*Mus musculus*	(17)
Ginno et al. 2020		Methylation and demethylation rates	*Mus musculus*	(1)
Hammoud et al. 2015	GSE62355	WGBS	*Mus musculus*	(18)
Kobayashi et al. 2012	DRA000484	MethylC-seq, WBA-seq	*Mus musculus*	(19)
Hill et al. 2018	GSE76973	WGBS	*Mus musculus*	(20)
Wang et al. 2018	GSE98151	WGBS	*Mus musculus*	(21)

Table S4. Logos for the position weight matrices (PWM) of the POU motifs used in this study.

Motif	PWM
CpGpal	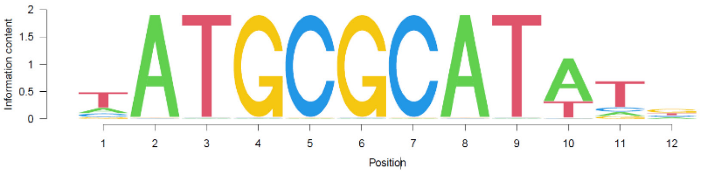
Octamer	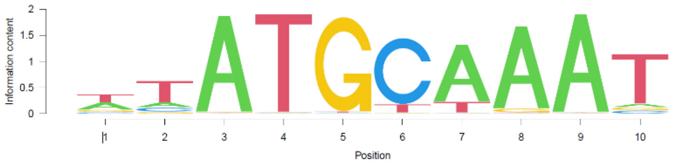
MORE	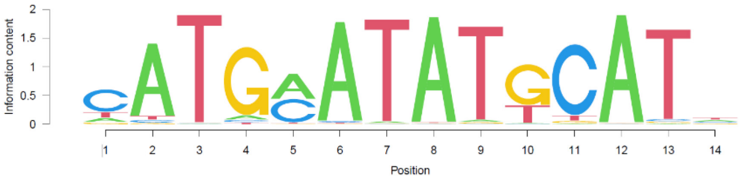
SoxOct	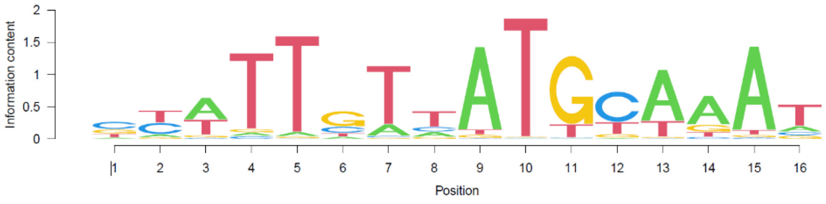

Table S5. PWMs of CpGpal from De Novo motif search (HOMER)

Study	Description	PWM	Rank	-logpvalue
Yin et al. 2017	DnmtTKO ESC	n/a	n/a	n/a
Yin et al. 2017	TetTKO ESC	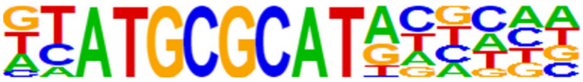	2	555
Yin et al. 2017	WT	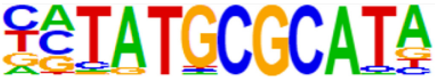	2	298
Knaupp et al. 2017	D3-Reprogramming	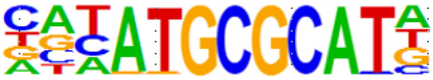	4	1871
Knaupp et al. 2017	D6-Reprogramming	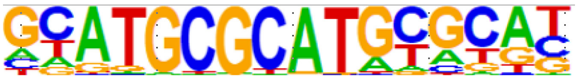	15	381
Knaupp et al. 2017	D9-Reprogramming	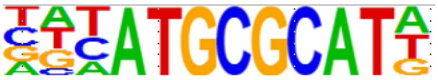	6	1252
Knaupp et al. 2017	D12-Reprogramming	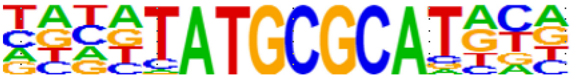	5	1573
Knaupp et al. 2017	iPSC	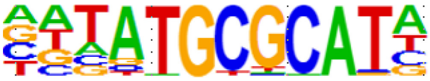	3	661
Chronis et al. 2017	OS D2-Reprogramming	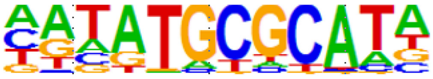	7	1054
Chronis et al. 2017	OSK D2-Reprogramming	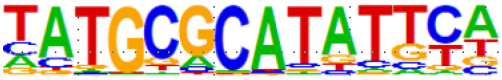	6	602
Chronis et al. 2017	OSKM D2-Reprogramming	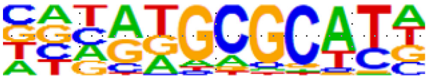	6	378
Chronis et al. 2017	Pre-IPSC	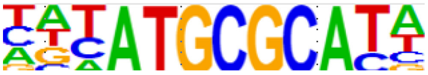	6	2059
Chronis et al. 2017	ESC	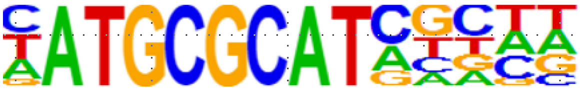	3	320
Shen et al. 2017	ESC	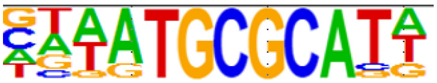	2	243
Zviran et al. 2018	D1-Reprogramming	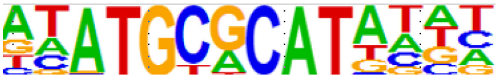	3	1400
Zviran et al. 2018	D2-Reprogramming	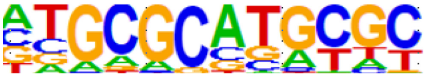	3	544

Study	Description	PWM	Rank	-log(pvalue)
Zviran et al. 2018	D3-Reprogramming	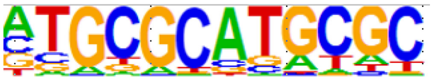	4	633
Zviran et al. 2018	D4-Reprogramming	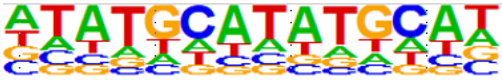	4	1799
Zviran et al. 2018	D5-Reprogramming	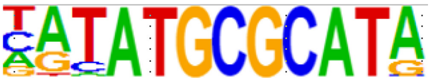	3	868
Zviran et al. 2018	D6-Reprogramming	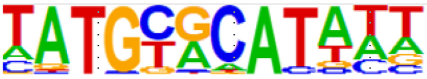	3	390
Zviran et al. 2018	D7-Reprogramming	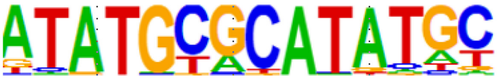	4	290
Zviran et al. 2018	D8-Reprogramming	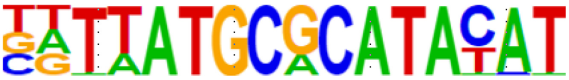	15	52
Zviran et al. 2018	ESC	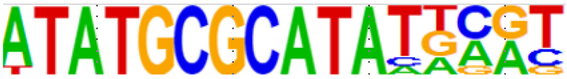	2	138
Adachi et al. 2018	EpiSC	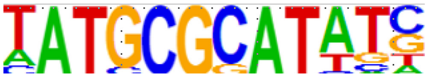	2	373
Adachi et al. 2018	8h EpiSC	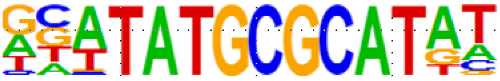	2	636
Adachi et al. 2018	24h EpiSC	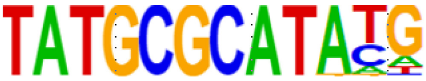	2	276
Adachi et al. 2018	48h EpiSC	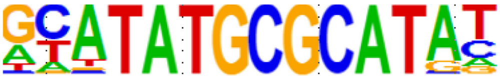	2	200
Adachi et al. 2018	72h EpiSC	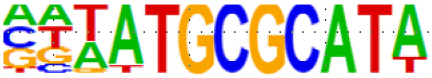	2	245
Malik et al. 2019	D1-Reprogramming	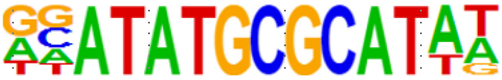	3	792
Malik et al. 2019	D3-Reprogramming	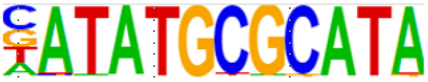	4	653
Malik et al. 2019	D5-Reprogramming	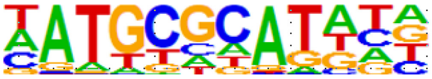	3	462
ENCODE	H1-hESC	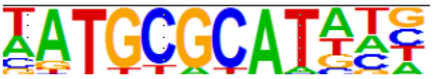	2	700

Table S6. PWMs of top motifs from *de novo* motif search (HOMER) in somatic POU datasets

Study	Description	PWM	-logpvalue
Shen et al.2017	Oct1 in ESCs Octamer	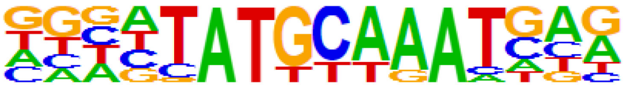	598
Shen et al.2017	Oct4 in ESCs SoxOct	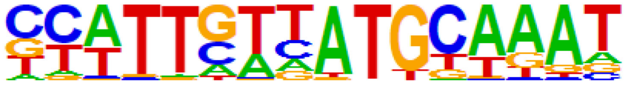	4152
Lodato et al. 2013	Brn2 in NPCs MORE	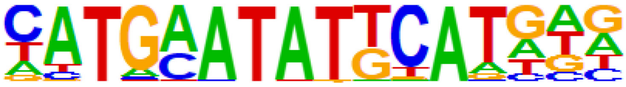	3129
Mistri et al. 2015	Brn1 in NSCs MORE	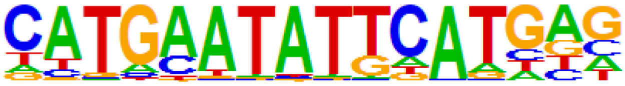	928
Mistri et al. 2015	Brn2 in NSCs MORE	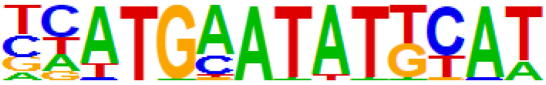	1277
Mistri et al. 2015	Oct6 in NSCs MORE	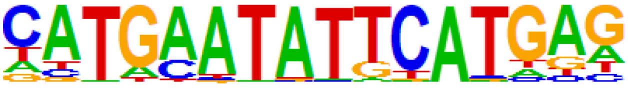	506
Heinz et al. 2010	Oct2 in B cells Octamer	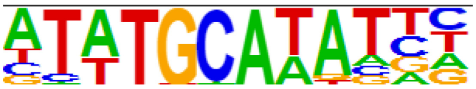	760

Table S7. Amino acid sequence of protein constructs used

Protein Construct	Amino Acid	Molecular Weight (kDa)
Oct4 POU	MGSSHHHHHHSSGLVPRGSHMPEESQDMKALQKELEQFAKLLKQ KRITLGYTQADVGLTLGVLFGKVFSQTTICRFEALQLSLKNMCKLRP LLEKWVEEADNNENLQEICKSETLVQARKRKRTSIENRVRWSLETM FLKCPKPSLQQITHIANQLGLEKDVVRVWFCNRRQKGKRSSIEYSQ RE	21.5
Oct4 POU_{HD}	GCKSETMVQARKRKRTSIENRVRWSLETMFLKCPKPSLQQITHIAN QLGLEKDVVRVWFCNRRQKGKRSSIEYSQRE	9.2
Brn2POU	GDPHSDEDTPTSDDLEQFAKQFKQRRIKLGFTQADVGLALGTLYG NVFSQTTICRFEALQLSFKNMCKLKPLLNKWLEEADSSSGSPTSIDK IAAQGRKRKKRTSIEVSVKGALESHFLKCPKPSAQEITSLADSLQLE KEVVRVWFCNRRQKEKRMTPPGGTL	18.4
Brn2 POU_{HD}	GKRKKRTSIEVSVKGALESHFLKCPKPSAQEITSLADSLQLEKEVVR VWFCNRRQKEKRMTPPGGTL	7.6
Brn2 POU_{S} linker	GDPHSDEDTPTSDDLEQFAKQFKQRRIKLGFTQADVGLALGTLYG NVFSQTTICRFEALQLSFKNMCKLKPLLNKWLEEADSSSGSPTSIDK IAAQGR	10.8

SUPPLEMENTARY METHODS

Specificity by Sequencing (Spec-seq)

The experiment was performed as described in (22,23). In brief, for the unmethylated library, combined DNA libraries (44bp) of degenerate Octamer motif sequences (ATGCNNNN, ATNNNNAT, NNNNTAAT) with 5' flanking sequence of GAGTCGTCTCGTCAGCAC and 3' flanking sequence of CCGTAGAGCACTCAGGTC for downstream processing were used. For the methylated library, flanks were redesigned to avoid CG dinucleotides 5'-GAT AGT CTC ATT TTC ACC TAG-ATGNNNAT -TTC CAT TAC AGT ATC TGT-3'. dsDNA libraries were generated with a FAM-labelled primer. The library was enzymatically methylated with CpG Methyltransferase (M.SssI; NEB # M0226S) and further digested by a cocktail of methyl-CpG–sensitive restriction enzymes from to remove unmethylated DNA (Hpa II , Hha I , Aci I, HpyCH4IV and BstU I; NEB # R0171S, R0139S, R0551S, R0619S,R0518S) (4). DNA libraries were subject to binding reactions with Oct4 DNA binding domain for 1 hour and subjected to an EMSA using an 9% PAGE/Agarose gel. After EMSA, the gels were stained with 3x GelRed® or visualsied using FAM, bands were excised and the DNA in the gel was extracted, and PCR purified. PCR was performed with primers compatible with Illumina adapter and contained different indexing barcodes (Illumina TruSeq Dual Index Library). A purified sample was then sent for NGS sequencing using HiSeq X Ten with Novogene Co., LTD.

Retrovirus production and IPSC reprogramming

Plat-E cells were seeded at 8 x 10^6 cells per 10 cm dish. 10 µg retroviral pMX vectors and 40 µg linear polyethyleneimine (Polysciences: #23966) dissolved in 1 mL plain DMEM were added to the cell after 16-24h for transfection. The medium was changed after 12-16 hours and virus-containing supernatants were collected at 48h and 72h after transfection, filtered through a 0.45-µm filter (Millipore), diluted to 12 mL with Plat-E medium, and mixed with 12 µL of 8mg/mL polybrene (Sigma-Aldrich: #40804ES76). OG2MEFs were seeded at a density of 1.5 x 10^4 per well for a 12-well plate and transduced on the next day with 0.5mL of each freshly harvested virus-containing supernatant. The second-round infection was done 24h later and after 24h the viral supernatant was replaced with mouse ESC medium (15% FBS, 1% GlutaMAX, 1% NEAA, 1% sodium pyruvate, 0.05mM β-mercaptoethanol, 50 µg/mL Vitamin C, 0.5% penicillin-streptomycin, 10 ng/mL LIF (prepared in-house) in DMEM). This day was defined as reprogramming day 0 and the medium was replaced daily. Cells were monitored and imaged using a fluorescence microscope (Olympus CKX53) during reprogramming. GFP^+ colonies were counted from the moment of appearance until day 13. Whole-well scans were taken using a Typhoon5 Biomolecular Imager (GE Healthcare).

Procedures for the generation of MEFs from mouse embryos were approved by the committee on the use of live animals in the teaching and research of HKU (CULATR, No. 5510-20).

Molecular Dynamics simulations

We solvated the systems in a truncated octahedron periodic box of SPCE water with the distance between any protein-DNA atom to the box edges larger than 11 Å. We added 62 neutralizing Na^+ ions and 150 mM KCl (225 K^+ and 225 Cl^- ions). For the ions we used the parameters developed by Li and Merz (24). We used the Amber-ff14SB (25) and the Amber-parmbsc1 (26) force fields for proteins and DNA respectively. We energy minimized and equilibrated each system with a stepwise protocol described previously (27) in which we applied restraints to keep the hydrogen bonds and hydrophobic interactions between DNA bases and between the POU_{HD} domains and the DNA bases.

With each model we performed 2 independent, 470 ns long MD simulations with periodic boundary conditions and a timestep of 2 fs by assigning different velocity distributions before the equilibration (after initial energy minimization). We used Langevin Dynamics (damping coefficient of 0.1 ps-1) and the Nose Hoover Langevin Piston method (period and decay of 1.2 and 1.0 ps respectively) to maintain the temperature at 300K and the pressure at 1 atm respectively. The direct calculation of the non-bonded interactions was truncated at 10 Å and the chemical bonds of hydrogens were kept rigid with the SHAKE algorithm. Long range electrostatics were calculated using the particle mesh Ewald algorithm. All simulations were performed in NAMD (28). Snapshots were selected for analysis every 5 ps.

To avoid the collapse of the freely moving POU_S domains on each other and on the POU_{HD} domains bound to DNA, we performed the simulations with restraints between each pair of two domains except the POU_{HD} - POU_{HD} pair. The minimal distance and coordination number between the Cα atoms of each pair of two domains were restrained to be larger than 15 and smaller than 1 respectively with a walled harmonic potential. The minimal distance, coordination number and the potential were used as defined in the COLVAR module of NAMD (colvars.github.io, (29)). The minimal distance was defined as generalized mean distance with a very high exponent (n=100) (see the Colvars manual for details).

SUPPLEMENTARY DATA

- **Supplementary Table S8.** Coordinates of Oct4/CpGpal sites combined from the three studies (7,9,17) as indicated in Figure S2C

- **Movie S1.** 470ns simulation of Oct4 bound to the CpGpal site at the center of a 40 bp genomic DNA sequence of gene - *Nr5a2,* in binding configuration 1 (Figure 6D) or "MORE like configuration". The POU_{HD} subdomain is specifically recognizing the AT steps at the 3' flanks of the CpGpal (5'-ATGCGCAT-3') while POU_S is detached and freely interacting with the DNA at the sides of the motif

- **Movie S2.** 470ns simulation of Oct4 bound to the CpGpal site at the center of a 40 bp genomic DNA sequence of Nr5a2 gene, in binding configuration 2 (Figure S7A). The POU_{HD} subdomain is specifically recognizing the AT step at the 5' flanks of the CpGpal (5'-ATGCGCAT-3') while POU_S is detached and freely interacting with the DNA at the sides of the motif

REFERENCES

1. Ginno, P.A., Gaidatzis, D., Feldmann, A., Hoerner, L., Imanci, D., Burger, L., Zilbermann, F., Peters, A., Edenhofer, F., Smallwood, S.A. *et al.* (2020) A genome-scale map of DNA methylation turnover identifies site-specific dependencies of DNMT and TET activity. *Nat Commun*, **11**, 2680.
2. Smallwood, S.A. and Kelsey, G. (2012) De novo DNA methylation: a germ cell perspective. *Trends Genet*, **28**, 33-42.
3. BabuRajendran, N., Palasingam, P., Narasimhan, K., Sun, W., Prabhakar, S., Jauch, R. and Kolatkar, P.R. (2010) Structure of Smad1 MH1/DNA complex reveals distinctive rearrangements of BMP and TGF-beta effectors. *Nucleic Acids Res*, **38**, 3477-3488.
4. Zuo, Z., Roy, B., Chang, Y.K., Granas, D. and Stormo, G.D. (2017) Measuring quantitative effects of methylation on transcription factor-DNA binding affinity. *Sci Adv*, **3**, eaao1799.
5. Malik, V., Glaser, L.V., Zimmer, D., Velychko, S., Weng, M., Holzner, M., Arend, M., Chen, Y., Srivastava, Y., Veerapandian, V. *et al.* (2019) Pluripotency reprogramming by competent and incompetent POU factors uncovers temporal dependency for Oct4 and Sox2. *Nat Commun*, **10**, 3477.
6. Malik, V., Zimmer, D. and Jauch, R. (2018) Diversity among POU transcription factors in chromatin recognition and cell fate reprogramming. *Cell Mol Life Sci*, **75**, 1587-1612.
7. Yin, Y., Morgunova, E., Jolma, A., Kaasinen, E., Sahu, B., Khund-Sayeed, S., Das, P.K., Kivioja, T., Dave, K., Zhong, F. *et al.* (2017) Impact of cytosine methylation on DNA binding specificities of human transcription factors. *Science*, **356**, eaaj2239.
8. Knaupp, A.S., Buckberry, S., Pflueger, J., Lim, S.M., Ford, E., Larcombe, M.R., Rossello, F.J., de Mendoza, A., Alaei, S., Firas, J. *et al.* (2017) Transient and Permanent Reconfiguration of Chromatin and Transcription Factor Occupancy Drive Reprogramming. *Cell Stem Cell*, **21**, 834-845 e836.
9. Zviran, A., Mor, N., Rais, Y., Gingold, H., Peles, S., Chomsky, E., Viukov, S., Buenrostro, J.D., Scognamiglio, R., Weinberger, L. *et al.* (2019) Deterministic Somatic Cell Reprogramming Involves Continuous Transcriptional Changes Governed by Myc and Epigenetic-Driven Modules. *Cell Stem Cell*, **24**, 328-341 e329.
10. Chronis, C., Fiziev, P., Papp, B., Butz, S., Bonora, G., Sabri, S., Ernst, J. and Plath, K. (2017) Cooperative Binding of Transcription Factors Orchestrates Reprogramming. *Cell*, **168**, 442-459 e420.
11. Sethi, A., Gu, M., Gumusgoz, E., Chan, L., Yan, K.K., Rozowsky, J., Barozzi, I., Afzal, V., Akiyama, J.A., Plajzer-Frick, I. *et al.* (2020) Supervised enhancer prediction with epigenetic pattern recognition and targeted validation. *Nat Methods*, **17**, 807-814.
12. Zhang, J., Lee, D., Dhiman, V., Jiang, P., Xu, J., McGillivray, P., Yang, H., Liu, J., Meyerson, W., Clarke, D. *et al.* (2020) An integrative ENCODE resource for cancer genomics. *Nat Commun*, **11**, 3696.
13. Shen, Z., Kang, J., Shakya, A., Tabaka, M., Jarboe, E.A., Regev, A. and Tantin, D. (2017) Enforcement of developmental lineage specificity by transcription factor Oct1. *Elife*, **6**.
14. Lodato, M.A., Ng, C.W., Wamstad, J.A., Cheng, A.W., Thai, K.K., Fraenkel, E., Jaenisch, R. and Boyer, L.A. (2013) SOX2 co-occupies distal enhancer elements with distinct POU factors in ESCs and NPCs to specify cell state. *PLoS Genet*, **9**, e1003288.
15. Mistri, T.K., Devasia, A.G., Chu, L.T., Ng, W.P., Halbritter, F., Colby, D., Martynoga, B., Tomlinson, S.R., Chambers, I., Robson, P. *et al.* (2015) Selective influence of Sox2 on POU transcription factor binding in embryonic and neural stem cells. *EMBO reports*, **16**, 1177-1191.
16. Heinz, S., Benner, C., Spann, N., Bertolino, E., Lin, Y.C., Laslo, P., Cheng, J.X., Murre, C., Singh, H. and Glass, C.K. (2010) Simple combinations of lineage-determining transcription factors prime cis-regulatory elements required for macrophage and B cell identities. *Mol Cell*, **38**, 576-589.
17. Adachi, K., Kopp, W., Wu, G., Heising, S., Greber, B., Stehling, M., Arauzo-Bravo, M.J., Boerno, S.T., Timmermann, B., Vingron, M. *et al.* (2018) Esrrb Unlocks Silenced Enhancers for Reprogramming to Naive Pluripotency. *Cell Stem Cell*, **23**, 266-275 e266.
18. Hammoud, S.S., Low, D.H., Yi, C., Lee, C.L., Oatley, J.M., Payne, C.J., Carrell, D.T.,

Guccione, E. and Cairns, B.R. (2015) Transcription and imprinting dynamics in developing postnatal male germline stem cells. *Genes Dev*, **29**, 2312-2324.

19. Kobayashi, H., Sakurai, T., Imai, M., Takahashi, N., Fukuda, A., Yayoi, O., Sato, S., Nakabayashi, K., Hata, K., Sotomaru, Y. *et al.* (2012) Contribution of intragenic DNA methylation in mouse gametic DNA methylomes to establish oocyte-specific heritable marks. *PLoS Genet*, **8**, e1002440.
20. Hill, P.W.S., Leitch, H.G., Requena, C.E., Sun, Z., Amouroux, R., Roman-Trufero, M., Borkowska, M., Terragni, J., Vaisvila, R., Linnett, S. *et al.* (2018) Epigenetic reprogramming enables the transition from primordial germ cell to gonocyte. *Nature*, **555**, 392-396.
21. Wang, C., Liu, X., Gao, Y., Yang, L., Li, C., Liu, W., Chen, C., Kou, X., Zhao, Y., Chen, J. *et al.* (2018) Reprogramming of H3K9me3-dependent heterochromatin during mammalian embryo development. *Nat Cell Biol*, **20**, 620-631.
22. Stormo, G.D., Zuo, Z. and Chang, Y.K. (2015) Spec-seq: determining protein-DNA-binding specificity by sequencing. *Brief Funct Genomics*, **14**, 30-38.
23. Tan, D.S., Chen, Y., Gao, Y., Bednarz, A., Wei, Y., Malik, V., Ho, D.H., Weng, M., Ho, S.Y., Srivastava, Y. *et al.* (2021) Directed Evolution of an Enhanced POU Reprogramming Factor for Cell Fate Engineering. *Mol Biol Evol*, **38**, 2854-2868.
24. Li, P., Song, L.F. and Merz, K.M., Jr. (2015) Systematic Parameterization of Monovalent Ions Employing the Nonbonded Model. *J Chem Theory Comput*, **11**, 1645-1657.
25. Maier, J.A., Martinez, C., Kasavajhala, K., Wickstrom, L., Hauser, K.E. and Simmerling, C. (2015) ff14SB: Improving the Accuracy of Protein Side Chain and Backbone Parameters from ff99SB. *J Chem Theory Comput*, **11**, 3696-3713.
26. Ivani, I., Dans, P.D., Noy, A., Perez, A., Faustino, I., Hospital, A., Walther, J., Andrio, P., Goni, R., Balaceanu, A. *et al.* (2016) Parmbsc1: a refined force field for DNA simulations. *Nat Methods*, **13**, 55-58.
27. Jerabek, S., Ng, C.K., Wu, G., Arauzo-Bravo, M.J., Kim, K.P., Esch, D., Malik, V., Chen, Y., Velychko, S., MacCarthy, C.M. *et al.* (2017) Changing POU dimerization preferences converts Oct6 into a pluripotency inducer. *EMBO reports*, **18**, 319-333.
28. Phillips, J.C., Braun, R., Wang, W., Gumbart, J., Tajkhorshid, E., Villa, E., Chipot, C., Skeel, R.D., Kale, L. and Schulten, K. (2005) Scalable molecular dynamics with NAMD. *J Comput Chem*, **26**, 1781-1802.
29. Fiorin, G., Klein, M.L. and Hénin, J. (2013) Using collective variables to drive molecular dynamics simulations. *Molecular Physics*, **111**, 3345-3362.
